# SYNPHONI: scale-free and phylogeny-aware reconstruction of synteny conservation and transformation across animal genomes

**DOI:** 10.1093/bioinformatics/btac695

**Published:** 2022-10-21

**Authors:** Nicolas Serge Matthieu Robert, Fatih Sarigol, Elisabeth Zieger, Oleg Simakov

**Affiliations:** Department of Neurosciences and Developmental Biology, University of Vienna, Vienna A-1030, Austria; Department of Neurosciences and Developmental Biology, University of Vienna, Vienna A-1030, Austria; Department of Evolutionary Biology, University of Vienna, Vienna A-1030, Austria; Department of Neurosciences and Developmental Biology, University of Vienna, Vienna A-1030, Austria

## Abstract

**Summary:**

Current approaches detect conserved genomic order either at chromosomal (macrosynteny) or at subchromosomal scales (microsynteny). The latter generally requires collinearity and hard thresholds on syntenic region size, thus excluding a major proportion of syntenies with recent expansions or minor rearrangements. ‘SYNPHONI’ bridges the gap between micro- and macrosynteny detection, providing detailed information on both synteny conservation and transformation throughout the evolutionary history of animal genomes.

**Availability and implementation:**

Source code is freely available at https://github.com/nsmro/SYNPHONI, implemented in Python 3.9.

**Supplementary information:**

Supplementary data are available at *Bioinformatics* online.

## 1 Introduction

Genes located on the same chromosome across species are considered syntenic [= ‘on the same ribbon’, (Renwick, 1971)]. A distinction is generally made between microsynteny (i.e. conservation of local/subchromosomal gene order) and macrosynteny (i.e. conserved synteny at chromosomal level). Both types of synteny are widely conserved across animal genomes (Irimia *et al.*, 2012; Simakov *et al.*, 2022), yet their functional significance remains elusive. While some microsyntenies are probably maintained because of constraints on the proximity between genes and their regulatory regions (Irimia *et al.*, 2012; Kikuta *et al.*, 2007; Zimmermann *et al.*, 2019), little is known about the potential evolutionary advantages of macrosynteny (Sun and Zhang, 2019; Véron *et al.*, 2011). Moreover, the distinction between micro- and macrosynteny can blur or even disappear in the context of 3D genomic structure and evolutionary modification. We thus developed SYNPHONI (detection of ancestral SYNteny based on PHylogeny and Ortholog Network Inference), a tool for detecting not only conserved synteny but also evolutionary changes to ancestral syntenic arrangements across all scales of metazoan genome organization and phylogenetic distance.

Many tools detect conserved microsyntenic blocks across multiple genomes [reviewed in (Lallemand *et al.*, 2020)], but most require stringent conservation of gene order (collinearity) [e.g. MCSCANX (Wang *et al.*, 2012), CYNTENATOR (Rödelsperger and Dieterich, 2010), AGORA (Nguyen, 2022), DESCHRAMBLER (Kim *et al.*, 2017) and i-ADHore (Proost *et al.*, 2012)]. Exceptions include the MicroSynteny tool (Simakov *et al.*, 2013), Gecko3 (Winter *et al.*, 2016) and EvolClust (Marcet-Houben and Gabaldón, 2019). However, these methods still require a priori specification of hard global thresholds on the number of intervening genes and syntenic block overlap. This imposes an artificial restriction on the scale at which synteny is considered informative, because it impedes the detection of blocks that are shared by smaller species subsets, blocks that underwent minor evolutionary modifications (e.g. expansions and translocations) or blocks that exceed a certain size [e.g. block shared by recently diverged taxa (Kaufmann and Frishman, 2014)].

In order to address the caveats outlined above, we designed SYNPHONI to work in a phylogeny-aware and scale-free manner. As such, SYNPHONI allows synteny reconstruction across different evolutionary distances, by outputting the syntenic complements of any given node of interest within the animal tree of life irrespective of collinearity or micro/macrosyntenic scale.

## 2 SYNPHONI algorithm

In SYNPHONI, a syntenic block is defined as a set of genes that are located in close proximity to each other (Supplementary Material, Step 2.5) in a given last common ancestor (LCA), without requiring collinearity. The descendants of each ancestral block are then recovered in extant genomes, regardless of whether their proximity has been maintained or not.

SYNPHONI was implemented in Python 3.9. The command-line interface software accepts as input a file containing groups of orthologs (OG), files containing gene coordinates and a species cladogram with phylogenetic clade names as node labels (polytomies allowed).

**Fig. 1.**
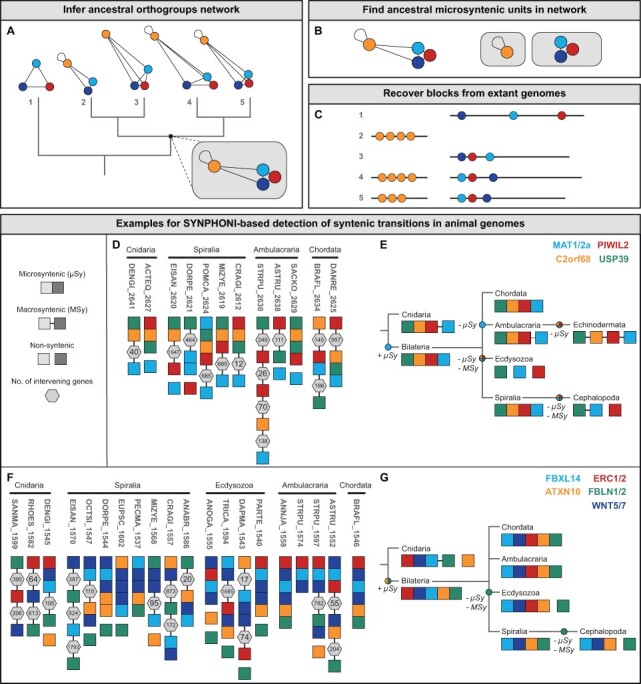
SYNPHONI-based detection of evolutionary transitions in genetic linkages across Metazoa. (**A–C**) Graphical summary of the SYNPHONI pipeline. Colored circles correspond to different OGs, edge lengths in the networks are proportional to intergenic distance. (**A**) A network of inter-OG minimum distances is built for every species of the taxon sample, in order to infer the state of the ancestral OG network in any node of interest. (**B**) Ancestral microsyntenic units are isolated from the network, by deleting edges above a distance threshold (see Supplementary Material). (**C**) Ancestral microsyntenic block are recovered from extant genomes, requiring only maintenance of synteny but not gene proximity (microsynteny). Each horizontal line corresponds to a chromosome, colored circles indicate genes, their color corresponds to their assigned OG. Distance between the circles corresponds to intergenic distance. (**D-G**) Colored squares represent members of the PWIL2 (**D, E**) and the WNT5/7 (**F, G**) syntenic blocks detected in the nephrozoan LCA, as indicated in the respective legends. Genes are shown as microsyntenic (µSy, contiguous squares), if they are separated by no more than 10 intervening genes. For macrosyntenic genes (MSy, squares connected by a line), the number of intervening genes is provided in gray hexagons. Genes that are not located on the same chromosome are shown as non-syntenic (noSy, spaced squares). (**D**) SYNPHONI-based characterization of the PWIL2 syntenic block for selected species (full dataset available in Supplementary Fig. S8), revealing frequent reshuffling of local gene order as well as species-specific losses of macro- or microsynteny. (**E**) Evolutionary scenario summarizing major syntenic transitions of the PIWIL2 cluster, including acquisition of MAT1/2 microsynteny in the last common ancestor of bilateria (+µSy) as well as microsynteny loss in echinoderms (−µSy) and macrosynteny loss in cephalopods and ecdysozoans (−MSy). (**F**) WNT5/7 gene cluster of selected species as identified by SYNPHONI (full dataset available in Supplementary Figs S8 and S9). As for the PIWIL2 cluster, reshuffling of local gene order and microsynteny losses are species-specific, whereas macrosynteny loss is gene-specific, indicating relaxed linkage constraints on FBLN1/2 and ATXN10. (**G**) Evolutionary scenario summarizing major syntenic transitions of the WNT5/7 cluster, such as acquisition of FBLN1/2 and ATXN10 microsynteny by the last common ancestor of bilateria (+µSy) as well as macrosynteny loss of FBLN1/2 in cephalopods and ecdysozoans (−MSy). Notably, these data indicate stronger linkage constraints on the evolutionary older microsyntenic relationships between WNT5/7, ERC1/2 and FBXL14, compared to the evolutionary younger relationships of ATXN10 and FBLN1/2. These examples demonstrate that SYNPHONI is a powerful tool for reconstructing the evolutionary history of genetic linkages at both micro- and macrosyntenic levels and across vast phylogenetic distances (A color version of this figure appears in the online version of this article)

The SYNPHONI algorithm proceeds as follows: build a matrix comprising the smallest number of intervening genes (*d*) between every possible pair of OGs (including self-pairs, i.e. syntenic paralogs) for all the species of the dataset. Only OG pairs that are syntenic in two or more species are kept. For each phylogenetic node of interest (*N*), we define its ingroup clades (e.g. Fig. 1A species 2/3 and 4/5 are descendants of *N*), its sister group (e.g. Fig. 1A species 5 is the sister group to the ingroup clades of *N*) and its outgroup (all species of the tree excluding the ingroup clades). The synteny of an OG pair (*OGi*, *OGj*) is stated in *N*, if it is detected as syntenic in a required minimum number of phylogenetic clades (see Supplementary Material for details).

SYNPHONI returns the number of phylogenetic clades (*k*) where an OG pair is syntenic. For each clade (*g*), the ancestral distance d*g*(*OGi-OGj*) is then calculated as the major mode of the kernel density estimate of the distances between all syntenic OGi and OGj pairs (Supplementary Material). The ancestral distance of *OGi* and *OGj* in the node of interest *N* (noted d*N*(*OGi*, *OGj*)) is thus defined as:

$$d_{N}({OG}_{i},{OG}_{j})\;=\;\frac{1}{k}\sum_{1}^{k}d_{g}(\mathrm{OGi},\mathrm{OGj})$$

Next, an OG network is built (raw graph), with edges stating synteny in *N* and with d*N* as an edge attribute. Only edges with d*N* < nmax are kept and the connected components are isolated. Details on the optimization of the nmax parameter can be found in the Supplementary Material. In brief, nmax ensures that SYNPHONI results respect the original definition of a syntenic block (Pevzner and Tesler, 2003) by allowing a more or less limited degree of micro/macrosyntenic rearrangements depending on the evolutionary time frame (depth of *N*). Each connected component of the trimmed graph is further decomposed to isolate maximal cliques in the raw graph. This yields refined OG sets corresponding to inferred ancestral microsyntenies present in *N*. Their descendants are recovered from extant genomes and the blocks sharing OGs are grouped into multi-species blocks. For final validation, the taxonomic composition of each multi-species block is assessed and only the blocks that were inherited from *N* are retained (Supplementary Material).

## 3 Insights and benchmarks

Running SYNPHONI on our dataset of 80 metazoan genomes (Supplementary Material), yielded 164 multispecies blocks in the metazoan LCA (Supplementary Fig. S4), 400 in the parahoxozoan LCA, 616 in the planulozoan LCA, 893 in the nephrozoan LCA, 807 in the protostome LCA and 1153 in the LCA of coleoid cephalopods. The analysis took <48 h using a single core.

For benchmarking, we compared SYNPHONI to the MicroSynteny tool (Simakov *et al.*, 2013; Robert *et al.*, 2022) and EvolClust (Marcet-Houben and Gabaldón, 2019), two methods that also detect non-collinear multispecies blocks across numerous genomes (Supplementary Material, Section S3). SYNPHONI provides the highest detection accuracy, as indicated by a significantly higher proportion of ‘core OGs’ (ancestrally microsyntenic OGs) per multispecies block (Wilcoxon rank-sum tests, *P* < 0.05, Supplementary Fig. S7B). Contrary to other methods, SYNPHONI outputs not only a single set of blocks for all input species but identifies the multispecies blocks of all nodes of interest. Furthermore, it does not require hard thresholds on block size or the number of intervening genes but detects synteny regardless of micro/macroscale. This results in a much greater detection sensitivity (as indicated by a significantly higher number of species per multispecies block, Wilcoxon rank-sum tests, *P* < 0.01, Supplementary Fig. S7C). More importantly, however, it allows detailed insights into how synteny evolved across different time scales. As such, SYNPHONI can be used to reconstruct both the emergence and all major lineage-specific transitions of syntenic blocks across Metazoa (e.g. Fig. 1D–G, Supplementary Figs S9 and S10).

Taken together, SYNPHONI is an efficient, accurate, phylogeny-aware and, most importantly, scale-free alternative to other methods, inferring microsyntenic complements of deep nodes within the animal tree of life.

## Supplementary Material

btac695_Supplementary_Data

## Data Availability

The data underlying this article are available in the article and in its Supplementary Material.
