# Supplementary material for: SYNPHONI: scale-free and phylogeny-aware reconstruction of synteny conservation and transformation across animal genomes
Source: Bioinformatics. 2022 Oct 21;38(24):5434–6. doi: 10.1093/bioinformatics/btac695 (PMC9750109; doi:10.1093/bioinformatics/btac695)
Supplement: btac695_Supplementary_Data [file btac695_supplementary_data.pdf]

SYNPHONI: Supplementary Information

Supplementary material for the publication: "SYNPHONI: scale-free & phylogeny-aware reconstruction of synteny conservation & transformation across animal genomes"

Nicolas Serge Matthieu Robert[1,*], Fatih Sarigol[1], Elisabeth Zieger[2] and Oleg Simakov[1,*]

[1]Department of Neurosciences and Developmental Biology, University of Vienna, Djerassiplatz 1, A-1030 Vienna, Austria, [2]Department of Evolutionary Biology, University of Vienna, Djerassiplatz 1, A-1030 Vienna, Austria.

*To whom correspondence should be addressed.

Abstract

Summary: Current approaches detect conserved genomic order either at chromosomal (macro-synteny) or at subchromosomal scales (microsynteny). The latter generally requires collinearity and hard thresholds on syntenic region size, thus excluding a major proportion of syntenies with recent expansions or minor rearrangements. "SYNPHONI" bridges the gap between micro- and macro-synteny detection, providing detailed information on both synteny conservation and transformation throughout the evolutionary history of animal genomes. **Availability and Implementation:** Source code is freely available 'here' {{https://github.com/nsmro/SYNPHONI}}, implemented in Python3.9.

Contact: nrobert.nicolas@orange.fr, oleg.simakov@univie.ac.at

1. Methods

1.1 Orthology assignment

Genome annotations and protein sequences of 80 species were obtained from the databases summarized in Supplementary Table 1. Only the longest isoform of each gene was retained. Orthogroups were inferred using OrthoFinder v2.4.1 (Emms and Kelly, 2019), in conjunction with diamond 0.9.36 (Buchfink *et al.*, 2015), and MCL 14.137 (Van Dongen, 2008). Identification of Phylogenetic Hierarchical Orthogroups at the root node (N0.HOGs) is based on orthogroup gene trees and species trees built using Mafft 7.427 (Katoh and Standley, 2013) and FastTree 2.1.11 (Price *et al.*, 2010). For all the following analyses, we consider genes belonging to the same N0.HOG to be orthologs.

Annotation of the proteomes of all species was done using eggNOG-mapper version 2 with default parameters (Huerta-Cepas *et al.*, 2017).

Supplementary Table 1: List of species used in this study, source of the proteins used for orthology and genome annotations for synteny analyses, and species prefix.

Full name	Source	Prefix
Acanthaster planci	NCBI (GCF_001949145.1_OKI-Apl_1.0)	ACAPL
Achatina fulica	Gigadb repository ftp://parrot.genomics.cn/gigadb/pub/10.5524/100001_101000/100647/	ACHFU
Acropora millepora	NCBI (GCF_004143615.1_amil_sf_1.1)	ACRMI
Actinia equina	ENSEMBL Rapid release ftp://ftp.ensembl.org/pub/rapid-release/ (actinia_equina_gca011057435)	ACTEQ
Adineta vaga	ENSEMBL Metazoa Release 47 (AMS_PRJEB1171_v1)	ADIVA
Amphimedon queenslandica	ENSEMBL Metazoa Release 47 (Aqu1)	AMPQU
Anadara broughtonii	Gigadb repository ftp://parrot.genomics.cn/gigadb/pub/10.5524/100001_101000/100607/	ANABR
Anneisia japonica	NCBI (GCF_011630105.1_ASM1163010v1)	ANNJA
Anopheles gambiae	Ensembl Metazoa release 45 (AgamP4.45)	ANOGA
Apis mellifera	NCBI (GCF_003254395.2_Amel_HAv3.1)	APIME
Apostichopus japonicus	NCBI (GCA_002754855.1_ASM275485v1)	APOJA
Acrchitheutis dux	Gigadb repository ftp://parrot.genomics.cn/gigadb/pub/10.5524/100001_101000/100676/	ARCDU
Asterias rubens	NCBI (GCF_902459465.1_eAstRub1.3)	ASTRU

Aurelia aurita	Online repository of Gold et al. 2019: https://doi.org/10.1038/s41559-018-0719-8) https://drive.google.com/drive/folders/1NC6bZ9cxWkZyofOsMPzrxIH3C7m1ySiu	AURAU
Branchiostoma floridae	data from Simakov et al. (https://doi.org/10.1038/s41559-020-1156-z)	BRAFL
Caenorhabditis elegans	ENSEMBL Metazoa Release 47 (WBcel235)	CAEEL
Callorhinchus milii	NCBI (GCF_000165045.1_Callorhinchus_milii-6.1.3)	CALMI
Canis lupus familiaris	NCBI (GCF_000002285.3_CanFam3.1)	CANLUFA
Capsaspora owczarzaki	ENSEMBL Metazoa Release 47 (capsaspora_owczarzaki_atcc_30864_gca_000151315)	CAPOW
Capitella teleta	ENSEMBL Metazoa Release 47 (Capitella_teleta_v1)	CAPTE
Chelonia mydas	NCBI (GCF_000344595.1_CheMyd_1.0)	CHEMY
Ciona intestinalis	GHOST database Hoya T line http://ghost.zool.kyoto-u.ac.jp/download_ht.html	CIOIN
Clytia hemisphaerica	MARIMBA (http://marimba.obs-vlfr.fr)	CLYHE
Corvus cornix cornix	NCBI (GCF_000738735.2_ASM73873v2)	CORCOCO
Crassostrea gigas	NCBI (GCF_902806645.1_cgigas_uk_roslin_v1)	CRAGI
Creolimax fragrantissima	Genome from NCBI (GCA_002024145.1_C_fragrantissima_v5) transcripts from GEO (GSE68616_Creolimax_fragrantissima) mapped with gmap	CREFR
Danio rerio	NCBI (GCF_000002035.6_GRCz11)	DANRE
Daphnia magna	NCBI (GCF_003990815.1_ASM399081v1)	DAPMA
Dendronephthya gigantea	NCBI (GCF_004324835.1_DenGig_1.0)	DENGI
Dorytheutis peallei	Schmidbaur et al. Submitted (b)	DORPE
Drosophila melanogaster	Ensembl Metazoa release 45 (BDGP6.22)	DROME
Eisenia andrei	National Genomics Data Center Genome Warehouse (ftp://download.big.ac.cn/gwh/Animals/Eisenia_andrei_Ean1_GWHACBE00000000/)	EISAN
Ephydatia muelleri	Online repository of Kenny et al 2020 (https://doi.org/10.1038/s41467-020-17397-w) (https://bitbucket.org/EphydatiaGenome/ephydatiagenome/downloads/)	EPHMU
Euprymna scolopes	Schmidbaur et al. Submitted (a)	EUPSC
Exaiptasia pallida	NCBI (GCF_001417965.1_Aiptasia_genome_1.1)	EXAPA
Folsomia candida	NCBI (GCF_002217175.1_ASM221717v1)	FOLCA
Gallus gallus	NCBI (GCF_000002315.6_GRCg6a)	GALGA

Helobdella robusta	ENSEMBL Metazoa Release 47 (Helro1)	HELRO
Hippocampus comes	NCBI (GCF_001891065.1_H_comes_QL1_v1)	HIPCO
Hofstenia miamia	Website of the Srivastava lab (http://srivastavalab.rc.fas.harvard.edu)	HOFMI
Hoilungia hongkongensis	Online repository of Eitel et al. 2018 (https://doi.org/10.1371/journal.pbio.2005359) https://bitbucket.org/molpalmuc/hoilungia-genome/src/master/tracks/	HOIHO
Homo sapiens	NCBI (GCF_000001405.39_GRCh38.p13)	HOMSA
Hydra vulgaris	NHGRI hydra2.0 (https://research.nhgri.nih.gov/hydra/)	HYDVU
Ixodes scapularis	NCBI (GCF_002892825.2_ISE6_asm2.2_deduplicated)	IXOSC
Latimeria chalumnae	NCBI (GCF_000225785.1_LatCha1)	LATCH
Lepisosteus oculatus)	NCBI (GCF_000242695.1_LepOcu1)	LEPOC
Lingula anatina	ENSEMBL Metazoa Release 47 (LinAna1.0)	LINAN
Lottia gigantea	ENSEMBL Metazoa Release 47 (Lotgi1)	LOTGI
Maylandia zebra	NCBI (GCF_000238955.4_M_zebra_UMD2a)	MAYZE
Mizuhopecten yessoensis	Provided by authors of Wang et al. 2017 (https://doi.org/10.1038/s41559-017-0120)	MIZYE
Mnemiopsis leidyi	NHGRI (https://research.nhgri.nih.gov/mnemiopsis/)	MNELE
Morbakka virulenta	OIST https://marinegenomics.oist.jp/morbakka_virulenta/download	MORVI
Mus musculus	NCBI (GCF_000001635.26_GRCm38.p6)	MUSMU
Nematostella vectensis	NCBI (GCF_000209225.1_ASM20922v1)	NEMVE
Octopus bimaculoides	NCBI (GCF_001194135.1_Octopus_bimaculoides_v2_0)	OCTBI
Octopus minor	Gigadb repository ftp://parrot.genomics.cn/gigadb/pub/10.5524/100001_101000/100503/	OCTMI
Octopus sinensis	NCBI (GCF_006345805.1_ASM634580v1)	OCTSI
Parasteoda tepidaorum	NCBI (GCF_000365465.2_Ptep_2.0)	PARTE
Pecten maximus	NCBI (GCF_902652985.1_xPecMax1.1)	PECMA
Pelodiscus sinensis	NCBI (GCF_000230535.1_PelSin_1.0)	PELSI
Penaeus vannamei	NCBI (GCF_003789085.1_ASM378908v1)	PENVA
Pleurobrachia bachei	NEUROBASE CDS and genome downloaded from https://neurobase.rc.ufl.edu cds mapped onto genome using gmap	PLEBA
Podarcis muralis	NCBI (GCF_004329235.1_PodMur_1.0)	PODMU
Pomacea caniculata	NCBI (GCF_003073045.1_ASM307304v1)	POMCA

Portunus tuberculatus	Gigadb repository ftp://parrot.genomics.cn/gigadb/pub/10.5524/100001_101000/100678/	PORTR
Priapulus caudatus	NCBI (GCF_000485595.1_Priapulus_caudatus-5.0.1)	PRICA
Ptychodera flava	OIST https://groups.oist.jp/molgenu/hemichordate-genomes	PTYFL
Ramazzotius varieornatus	NCBI (GCA_001949185.1_Rvar_4.0)	RAMVA
Rhopilema esculentum	Proteins from Gigadb repository (ftp://parrot.genomics.cn/gigadb/pub/10.5524/100001_101000/) mapped onto NCBI genome (GCA_013076305.1_ASM1307630v1) with gmap	RHOES
Saccoglossus kowalevskii	OIST https://groups.oist.jp/molgenu/hemichordate-genomes	SACKO
Salpingoeca rosetta	ENSEMBL Metazoa Release 47 (salpingoeca_rosetta_gca_000188695)	SALRO
Sanderia malayensis	genome from NCBI (GCA_013076295.1_ASM1307629v1) annotation from online repository (https://github.com/nongwy/JellyfishGenomeData/raw/master)	SANMA
Schmidtea mediterranea	PLANMINE http://planmine.mpi-cbg.de (genome: dd_Smes_g4 annotation: smes_v2_hconf_SMESG)	SCHME
Sphaeroforma arctica	NCBI (GCF_001186125.1_Spha_arctica_JP610_V1)	SPHAR
Strigamia maritima	ENSEMBL Metazoa Release 47 (Smar1)	STRMA
Strongylocentrotus purpuratus	NCBI (GCF_000002235.5_Spur_5.0)	STRPU
Sycon ciliatum	CDS and genome downloaded from https://datadryad.org/stash/dataset/doi:10.5061/dryad.tn0f3 cds mapped onto genome using gmap	SYCCI
Trichoplax adhaerens	ENSEMBL Metazoa Release 47 (ASM15027v1)	TRIAD
Tribolium castaneum	ENSEMBL Metazoa Release 47 (Tcas5.2)	TRICA
Xenopus laevis	NCBI (GCF_001663975.1_Xenopus_laevis_v2)	XENLA

1.2 Detection of microsyntenic blocks

The MicroSynteny tool (Simakov *et al.*, 2013), EvolClust (Marcet-Houben and Gabaldón, 2019), and SYNPHONI (this article) were used to detect microsyntenic blocks in our 80-species sample (Supplementary Table 1). The MicroSynteny tool was run with the same parameters as in (Robert *et al.*, 2022). For EvolClust, three thresholds had to be specified a priori:

- Minimal block size = 3 genes (--minSize 3),
- Maximal block size = 80 genes (--maxSize 80)
- Maximal number of intervening genes between two consecutive genes = 30 (--non_homologs 30)

These parameters are less stringent than the defaults (Marcet-Houben and Gabaldón, 2019). They were chosen for comparability with SYNPHONI, which also requires a minimal block size of at least 3 genes. Furthermore, blocks detected by SYNPHONI generally comprised less than 80 genes (67716/67718) and the highest number of intervening genes before splitting a block was determined to be 29 based on dynamic estimation (Supplementary Figure 3, Supplementary Table 4). A detailed description of the SYNPHONI pipeline and its parameters is provided in section 2.

1.3 Taxonomic classification of microsyntenic blocks

In contrast to SYNPHONI which outputs the microsyntenic complements of each node of interest (N), other methods return only a single set of conserved microsyntenic blocks for the entire taxonomic sample. We therefore classified MicroSynteny tool and EvolClust blocks according to their phylogenetic distribution, using our **BlocksByNode.py** script (Supplementary Table 3). This script determines whether a multispecies block is present in a given N, based on which species possess block homologs. To this end, phylogenetic clades were defined as follows:

- Ingroup = all species descended from a given N
- Sister group = all the species of the clade with the closest relationship to the ingroup
- Outgroup = all species outside of a given ingroup
- Ingroup clade = all species of a children clade within a given ingroup
- Outgroup clade = all species of a children clade within a given outgroup

The cladogram used for defining the phylogenetic clades of each N (i.e. Metazoa, Parahoxozoa, Planulozoa, Nephrozoa, Protostomia and Cephalopoda/coleoid cephalopods) can be found in Supplementary Figure 1. A clade is considered “populated” by a multispecies block, if the block is found in a number of species equal to or higher than a threshold *m* (or in all species of the clade, if it comprises less than *m* species). The *m* thresholds used for analyzing MicroSynteny tool and EvolClust results are summarized in Supplementary Table 2. A block is considered present in N, if it populates at least two ingroup clades of N or both an ingroup and an outgroup clade.

1.4 Core orthogroup detection for block verification

To test whether a detected block is evolutionarily meaningful, we developed the concept of core orthogroups (core OGs), which are OGs that were inherited from the ancestral multispecies block. Blocks comprising no core OGs were classified as noise (Supplementary Figure 7). When comparing blocks across methods, we considered only blocks comprising at least three distinct core OGs (Supplementary

Figure 8), since EvolClust does not detect blocks composed of paralogs. To determine whether the presence of an OG within a given multispecies block is ancestrally conserved, we assessed its taxonomic distribution using our **describe_coreOGs.py** script (Supplementary Table 3). This script considers an OG as "populating" a given phylogenetic clade (see section 1.3), if it is found in a number of species equal to or higher than a threshold *m* (or in all species of the phylogenetic clade, if it comprises less than *m* species). For a given node and multispecies block, an OG is thus classified as a core OG, if it meets one of the following conditions:

- The OG populates at least two ingroup clades
- The OG populates one ingroup clade and at least one sister group
- The OG populates one ingroup clade and the outgroup

For each node of interest, the *m* parameters used to identify the core OGs of the blocks detected by the MicroSynteny tool, EvolClust and SYNPHONI are summarized in Supplementary Table 2.

Supplementary Table 2. Species thresholds (*m*) used to classify multispecies blocks by their node of origin and to detect core OGs. A cladogram depicting the different nodes can be found in Supplementary Figure 1. The species_threshold parameters shown are used by the **BlocksByNode.py** script and the **describe_OGs.py** script (Supplementary Table 3).

	Metazoa	Parahoxozoa	Planulozoa	Nephrozoa	Protostomia	Cephalopoda
**MicroSynteny**	2	2	2	2	2	2
**SYNPHONI**	2	2	2	2	2	2
**EvolClust**	1	1	1	1	1	1

Supplementary Table 3. Links to the scripts used in this study.

**Script name**	**Link to script**
BlocksByNode.py	https://github.com/nsmro/comparative_genomics_utils/blob/main/Microsynteny/BlocksByNode.py
describe_OGs.py	https://github.com/nsmro/comparative_genomics_utils/blob/main/Microsynteny/describe_ogs.py
step2.5_optimal_nmax.py	https://github.com/nsmro/synphoni/blob/main/tools/step2.5_optimal_nmax.py
analysis_intervening_genes.py	https://github.com/nsmro/synphoni/blob/main/analysis_intervening_genes.py+

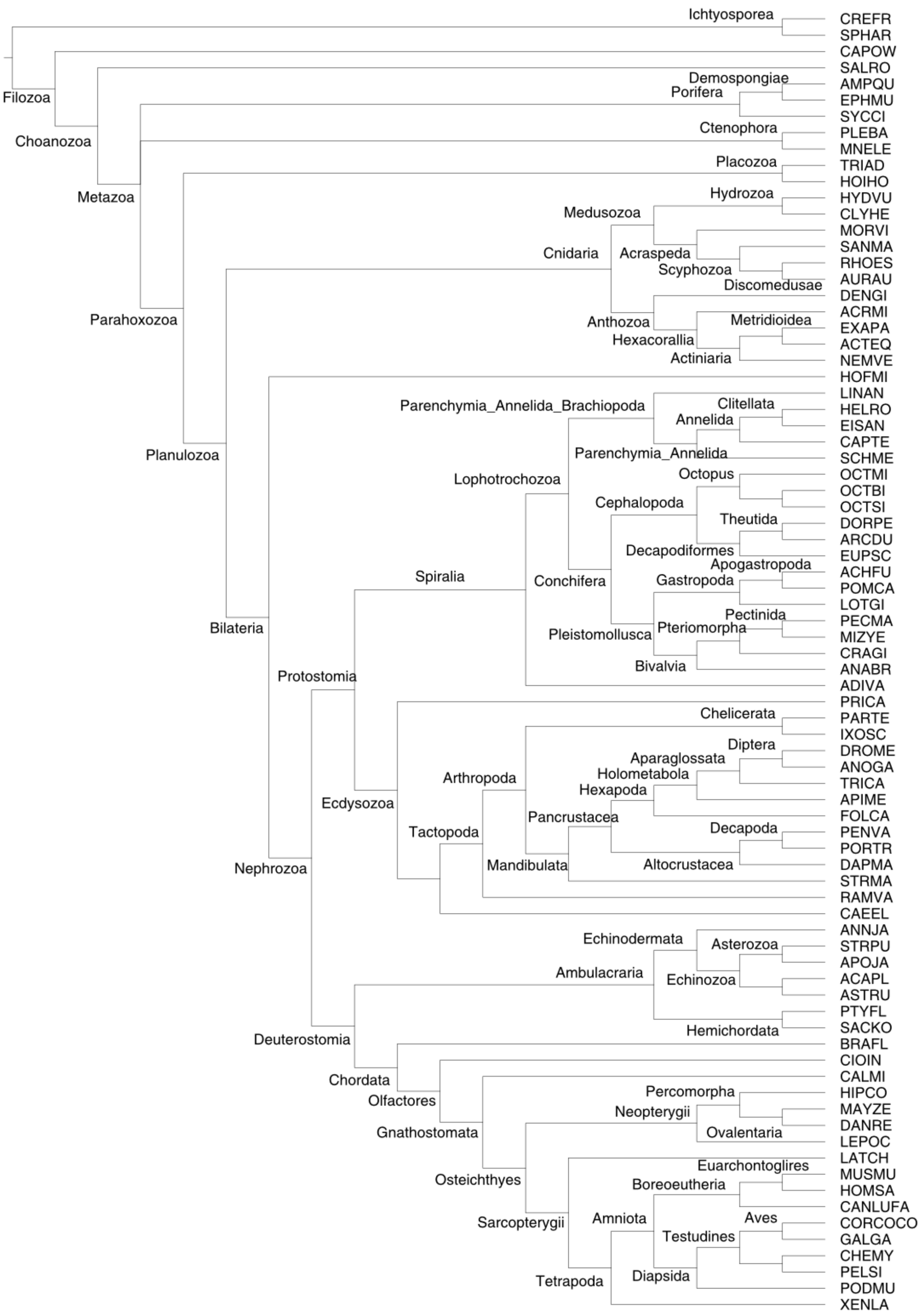

Ichtyosporea
Filozoa
Choanozoa
Demospongiae
Porifera
Ctenophora
Placozoa
Metazoa
Hydrozoa
Medusozoa
Cnidaria
Acraspeda
Scyphozoa
Discomedusae
Parahoxozoa
Anthozoa
Metridioidea
Hexacorallia
Actiniaria
Parenchymia_Annelida_Brachiopoda
Clitellata
Annelida
Parenchymia_Annelida
Planulozoa
Lophotrochozoa
Octopus
Cephalopoda
Theutida
Decapodiformes
Apogastropoda
Spiralia
Conchifera
Gastropoda
Pectinida
Bilateria
Pleistomollusca
Pteriomorpha
Bivalvia
Protostomia
Chelicerata
Diptera
Aparaglossata
Holometabola
Arthropoda
Hexapoda
Ecdysozoa
Pancrustacea
Decapoda
Tactopoda
Nephrozoa
Mandibulata
Altocrustacea
Echinodermata
Asterozoa
Ambulacraria
Echinozoa
Hemichordata
Deuterostomia
Chordata
Olfactores
Percomorpha
Neopterygii
Gnathostomata
Ovalentaria
Euarchontoglires
Osteichthyes
Boreoeutheria
Amniota
Aves
Sarcopterygii
Testudines
Diapsida
Tetrapoda
CREFR
SPHAR
CAPOW
SALRO
AMPQU
EPHMU
SYCCI
PLEBA
MNELE
TRIAD
HOIHO
HYDVU
CLYHE
MORVI
SANMA
RHOES
AURAU
DENGI
ACRMI
EXAPA
ACTEQ
NEMVE
HOFMI
LINAN
HELRO
EISAN
CAPTE
SCHME
OCTMI
OCTBI
OCTSI
DORPE
ARCDU
EUPSC
ACHFU
POMCA
LOTGI
PECMA
MIZYE
CRAGI
ANABR
ADIVA
PRICA
PARTE
IXOSC
DROME
ANOGA
TRICA
APIME
FOLCA
PENVA
PORTR
DAPMA
STRMA
RAMVA
CAEEL
ANNJA
STRPU
APOJA
ACAPL
ASTRU
PTYFL
SACKO
BRAFL
CIOIN
CALMI
HIPCO
MAYZE
DANRE
LEPOC
LATCH
MUSMU
HOMSA
CANLUFA
CORCOCO
GALGA
CHEMY
PELSI
PODMU
XENLA

Supplementary Figure 1. Cladogram used to determine the phylogenetic distribution of multispecies blocks. Binomial names corresponding to the species prefixes are listed in Supplementary Table 1. Relationships outside of Metazoa are based on **(Torruella *et al.*, 2012)**. Relationships between major metazoan phyla are reviewed in **(Dunn *et al.*, 2014)**. Position of Xenacoelomorpha as a sister group to Nephrozoa is based on **(Cannon *et al.*, 2016)**. Relationships within Arthropoda are based on **(Giribet and Edgecombe, 2017)**. Relationships within Spiralia are based on **(Marlétaz *et al.*, 2019)**. Relationships within Echinodermata are based on **(Reich *et al.*, 2015)**. Relationships within Amniota are based on **(Chiari *et al.*, 2012)**. Relationships within Cnidaria are based on **(Kayal *et al.*, 2018)**.

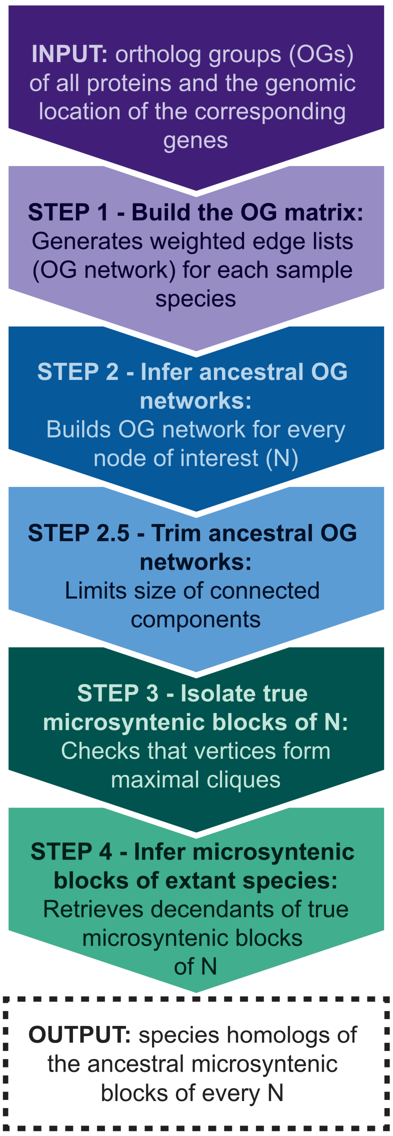

Supplementary Figure 2. Summary of the workflow of the SYNPHONI pipeline.

2. Detailed description of the SYNPHONI pipeline and rationale

The SYNPHONI pipeline comprises four main stages (Supplementary Figure 2) and requires as input the genomic coordinates of all genes as well as a list of all orthologous gene groups (orthogroups, OGs). Since only the smallest intergenic distance between two syntenic OGs is considered in our analysis, a fine-grained orthology is needed to detect as many inter-OG distances as possible. Thus, we used the phylogenetic hierarchical orthogroups of the root node (N0.HOGs) that are inferred by OrthoFinder version 2.4 (Emms and Kelly, 2019) or higher.

STEP 1. Build the orthogroup matrix.

The aim of this step is to measure the minimum intergenic distance between every possible pair of OGs that show conserved synteny in a given number of species. The distances between syntenic self pairs (paralogs belonging to the same orthogroup (OG)) are also measured.

Two parameters can be adjusted to limit the number of pairs of OGs returned:

- *species_threshold* = the minimum number of species in which an OG pair must be syntenic to be retained. The default is 2, according to the definition of conserved synteny.
- *max_para* = maximum number of paralogs per OG and species. If an OG comprises more paralogs than specified for any given species, it will be discarded from further analysis. Default is set to 100, to exclude highly duplicated orthologs or promiscuous orthologs that have been erroneously assigned to the same N0.HOG.

This preparatory step accounts for the majority of the runtime (24 hours out of the 48 hours used for detecting the blocks of six different nodes in our sample of 80 species).

The number of base pairs is not a suitable measure for comparing intergenic distances between species, since animal genome sizes vary greatly even within the same phylum (Wong *et al.*, 2019; Murgarella *et al.*, 2016). However, the linear relationship between assembly size and the reciprocal of gene density (i.e. assembly size / total number of genes) suggests that the number of intervening genes is independent of genome size (Robert *et al.*, 2022). Thus, SYNPHONI measures the distance between two syntenic orthologs based on the number of intervening genes between them.

For a given OG pair (OG_i and OG_j), SYNPHONI only records the shortest possible distance between any member of OG_i and any member of OG_j. Given the pervasiveness of gene duplication across animal phyla (Fernández and Gabaldón, 2020; Paps and Holland, 2018; Srivastava *et al.*, 2010; Leclère *et al.*, 2019; Sacerdot *et al.*, 2018), we make the assumption that the shortest distance will generally reflect the ancestral distance (i.e. positional orthologs (Dewey, 2011)).

Syntenic paralogous gene pairs (OG self pairs) could either emerge through subchromosomal duplication (duplication of a chromosome segment) or whole genome duplication. Since the latter typically results in the emergence of non-syntenic paralogs (Sacerdot *et al.*, 2018), a secondary interchromosomal

rearrangement would be required to give rise to syntenic paralogs. Thus, the more parsimonious scenario is that syntenic paralogs emerged by subchromosomal duplication. A majority of the paralogs that emerged by subchromosomal duplication are located in close proximity to each other (reviewed in (Mendivil Ramos and Ferrier, 2012)). Accordingly, we assume that the shortest distance between paralogous genes corresponds to their ancestral intergenic distance.

The matrix outputted by this step contains a weighted edge list (OG graph) for each species, which comprises the minimum intergenic distances between all possible pairs of syntenic OGs. Each OG graph thus corresponds to a “species OG network”, where the OGs are vertices, the edges are syntenic relationships, and the edge weights are the minimum distances between two syntenic OGs of a given species.

STEP 2. Infer node specific orthogroup networks.

This step of the SYNPHONI pipeline infers ancestral OG networks from the species OG networks measured in step 1. For every OG pair inferred as syntenic in a taxonomic node of interest (N), an ancestral distance will be returned.

The required input includes the OG matrix created by step 1, a species cladogram with node names (polytomies allowed, e.g. Supplementary Figure 1) and a list of the node names for which the ancestral OG networks should be inferred.

For every N, we define ingroup clades, sister group(s) and the outgroup. Ingroup clades are the children clades of N (at least two, more if N is polytomic). Sister groups are all the children clades of the parent node of N that are not ingroups (no sister group if N is the root node, one sister group if the parent node of N is binary, more than one sister group if the parent node of N is polytomic). The outgroup encompasses all the species that are not part of any ingroup clades (no outgroup if N is the root node).

One parameter can be used to influence the sensitivity of detection:

- *species_threshold* (*m*) = minimum number of species of a phylogenetic clade (ingroup clade, sister group or outgroup, see below) that must possess a syntenic OG pair for it to be considered as “populating” the phylogenetic clade. If a clade is smaller than *m*, it is considered “populated” by an OG pair, if that OG pair is syntenic in all species of the clade. Thus, *m* is an integer equal to or greater than 1.

A syntenic OG pair is considered as “populating” a phylogenetic clade, if it is found in at least *m* species of the clade (or in all species, if the clade comprises less than m species). In order to be retained, a syntenic OG pair must meet one of the following conditions:

1. It populates at least two ingroup clades
2. It populates at least one ingroup clade and at least one sister group
3. It populates at least one ingroup clade and the outgroup

For every syntenic OG pair (OG_i,OG_j) that is retained, the first condition satisfied determines which phylogenetic clades (g) will be used for inferring the ancestral distance between OG_i and OG_j in N (noted $d_N(OG_i, OG_j)$).

For each phylogenetic clade (*g)*, we first calculate the ancestral distance between OG_i and OG_j (noted $d_g(OG_i, OG_j)$). To this end, the distances measured between OG_i and OG_j are recovered for all species of a given g. The obtained sample of distances in *g* follows a probability density function (PDF_g) that is estimated by a gaussian kernel density estimate. We define d_g as the value of distance corresponding to the global maxima of the estimate of PDF_g.

The ancestral distance between OG_i and OG_j in N ($d_N(OG_i, OG_j)$) is thus defined as the mean of all d_g estimates ($d_g(OG_i, OG_j)$), with k corresponding to the number of phylogenetic clades that are populated by the syntenic OG pair (OG_i,OG_j):

$$d_N(OG_i, OG_j) \; = \frac{1}{k}\sum_{1}^{k} \quad d_g(OGi, OGj)$$

For every node N, the result of step 2 can be represented as a graph (G_N), where OGs are vertices and their edges are ancestral syntenic relationships with the inferred d_N values as edge weights. Self loops represent cases where at least two paralogs of the OG are ancestrally syntenic.

From our taxonomic sample of 80 species, six nodes of interest (N) were selected for analysis (Supplementary Table 1, Supplementary Figure 1). For every N, increasing the *species_threshold m* leads to a network with less vertices, which means that less OGs are retained (Supplementary Table 4). Accordingly, *m* can be viewed as a stringency value. If *m* is larger than the number of species within a phylogenetic clade (e.g. ingroup/sister group/outgroup), syntenic relationships must be found in all species of this clade in order to be retained. Given the pervasiveness of gene loss (Albalat and Cañestro, 2016) and synteny loss (Winter *et al.*, 2016), this is not a reasonable requirement. We thus recommend choosing an *m* value that is lower than the minimum number of species per phylogenetic clade. Conversely, an *m* of 1 improves detection sensitivity (higher OG retention, Supplementary Table 4), but also increases the likelihood of false positives (i.e. detection of syntenic relationships that emerged convergently, or result from errors in orthology assignment). To account for this, the *m* value chosen for inferring the ancestral OG networks of Metazoa, Parahoxozoa, Protostomia and Cephalopoda was of two. Given the larger size of the ingroup clades of Nephrozoa and Planulozoa (Supplementary Figure 1), an *m* of three was selected for these nodes.

Supplementary Table 4. Number of OGs in the ancestral OG networks (G_N) inferred for different nodes (columns) with varying species thresholds *m* (rows). The m values that were chosen for the analyses presented in this article are underlined and in bold.

m	Metazoa	Parahoxozoa	Planulozoa	Nephrozoa	Protostomia	Cephalopoda
**1**	17228	18441	24451	29056	28031	14575
**2**	**<u>6621</u>**	**<u>7601</u>**	10436	13533	**<u>12691</u>**	**<u>10178</u>**
**3**	3841	5451	**<u>8179</u>**	**<u>10774</u>**	10156	7271
**4**	3482	4899	6976	9528	9244	7038
**5**	3233	4598	5966	8607	8443	6795
**6**	3057	4335	5027	7745	7509	6505

STEP 2.5. Trimming of the ancestral OG network

The original definition of a syntenic block states that it can be subject to micro-rearrangements but is interrupted by macro-rearrangements (Pevzner and Tesler, 2003).

The SYNPHONI pipeline identifies ancestral microsyntenic blocks, i.e. sets of genes that were located in close proximity to each other in a node of interest (N), but might have undergone independent micro-rearrangements in the different lineages descended from N. In an ancestral OG network (G_N), this is represented by a set of connected vertices (OGs) that can be visited via a path where all the edge weights (ancestral intergenic distances, d_N) do not exceed a distance threshold *nmax*. This is equivalent to identifying connected components in a trimmed graph GT_N that comprises all the vertices of G_N, but only includes edges with an edge weight below the *nmax* threshold. The nmax threshold thus restricts the maximum number of intervening genes allowed between ancestrally syntenic OGs within GT_N:

- *nmax* threshold = maximum edge weight d_N allowed in GT_N. All edges of G_N with d_N> *nmax* will be absent from GT_N.

The choice of nmax will restrict the overall size of the ancestral microsyntenic blocks/OG sets detected by SYNPHONI, since syntenic OGs connected by an edge with a weight above *nmax* will be considered split in N. It is thus important to identify the best-suited *nmax* for trimming the ancestral OG network of each N (Supplementary Figure 1). To this end, we measured the proportion of vertices (OGs) in the largest component of GT_N with varying *nmax* values (Supplementary Figure 3).

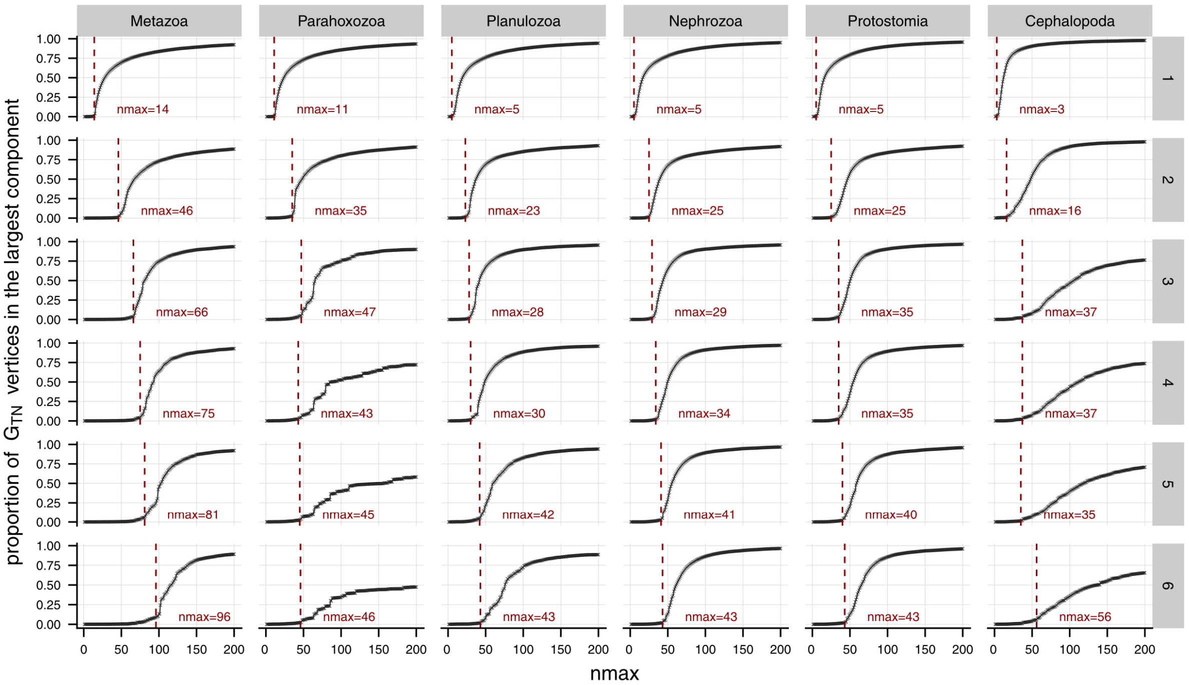

Supplementary Figure 3. Proportion of vertices (OGs) that are located within the largest component of the trimmed ancestral OG networks (GT_N), as a function of the distance threshold *nmax*. Columns correspond to the nodes of interest (N), for which GT_Ns have been inferred. Rows correspond to varying species_threshold values (*m,* left side) that were used to infer ancestral OG networks (G_N) in step 2 of the SYNPHONI pipeline. Dashed red vertical lines indicate the lower shoulder of the sigmoid curve, which indicates the optimal *nmax* threshold for the corresponding GT_N. The optimal *nmax* is thus defined as the x-left value and determined using the Extremum Distance Method **(Christopoulos, 2016)**.

Interestingly, the proportion of vertices within the largest component of GT_N (%GT_N), when expressed as a function of *nmax,* follows a sigmoid curve, regardless of the node of interest (N) and the species_threshold (*m*) chosen in step 2 (Supplementary Figure 3). If *nmax* is below 10, the largest component comprises less than 5% of the total number of vertices of GT_N. Conversely, if *m* is 1 and *nmax* is above 180, the largest component comprises almost 100% of the vertices of GT_N (Supplementary Figure 3). This is likely because of OGs with paralogs located on multiple chromosomes.

The %GT_N values can be used to identify at which *nmax* value connected components likely correspond to ancestral microsyntenic blocks rather than noise or ancestral macrosynteny. In the LCA of Nephrozoa, the largest ancestral linkage group (ALG ”A1”) comprises 11% of the total number of ancestrally macrosyntenic OGs (Simakov *et al.*, 2022). Thus, in order to resolve subchromosomal linkage groups (i.e. microsyntenic blocks), %GT_N must be lower than 0.11 (= all 261 OGs of the largest ALG (ALG “A1”) divided by all 2369 ancestrally microsyntenic OGs (Simakov *et al.*, 2022)). Otherwise, the detected OG sets would be too large (as indicated by the sigmoid curve reaching its exponential phase, Supplementary Figure 3) and would mostly correspond to ALGs (ancestral macrosynteny) or noise, instead of ancestral microsyntenic blocks. The best-suited *nmax* is hence located on the left shoulder of the sigmoid curve (Supplementary Figure 3), where %GTN is as high as possible (to avoid splitting large

ancestrally microsyntenic OG sets) without entering into the exponential phase (to avoid ALG/noise detection). In order to make the identification of this point easy to replicate and consistent across all N and *m* settings, we used the Extremum Distance Method (Christopoulos, 2016). We include a python implementation of this method in the SYNPHONI package (**step2.5_optimal_nmax.py**, Supplementary Table 3).

The optimal *nmax* for our sample, as determined by the above-described method, is inversely correlated to the number of vertices (OGs) within G_N, showing that it is independent of the evolutionary age of N.

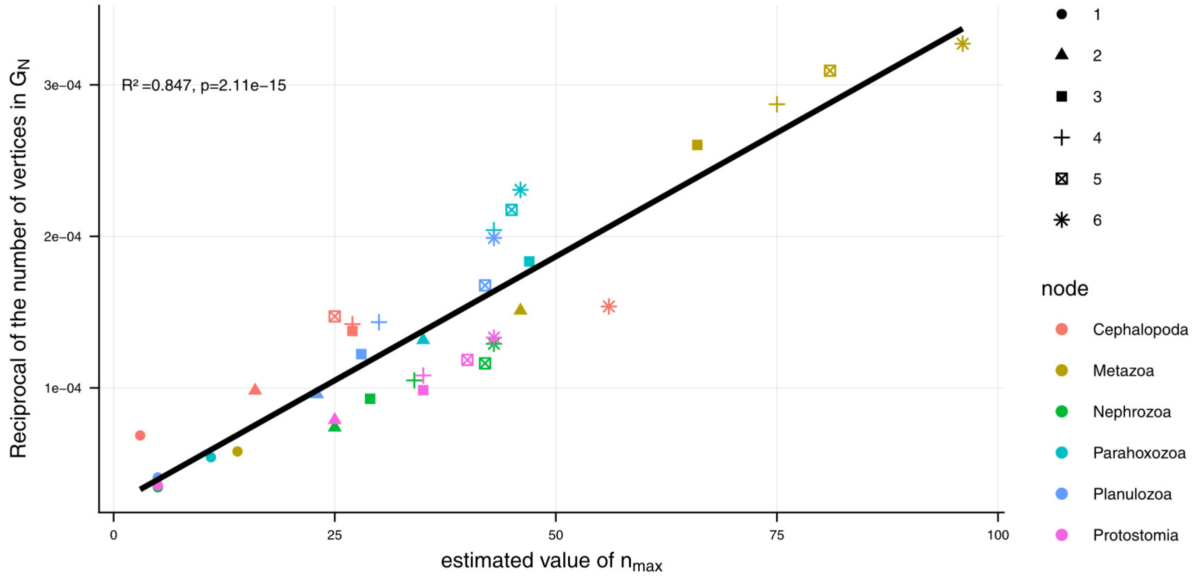

Supplementary Figure 4. The optimal *nmax* is inversely correlated to the number of vertices (OGs) in G_N. The R^2 coefficient and p-values are indicated in the plot. As indicated in the legend (left), shapes and colors of the points correspond to the species_threshold (*m*) chosen in step 2 and to the node (N) of G_N, respectively.

STEP 3. Isolate ancestrally microsyntenic OG sets

Every connected component isolated from GT_N should correspond to a set of OGs "C" that are ancestrally microsyntenic in the node of interest (N). Our next goal is to confirm whether all the OGs of C do indeed belong to a "true" microsyntenic block of N. This is necessary since OGs located on different chromosomes could be assigned to C, if they share ancestral microsynteny with paralogs of at least one OG of C. To avoid merging microsyntenic blocks from different chromosomes that overlap due to paralogs, we can analyze the induced subgraph of G_N ($G_N[C]$), which comprises all the edges linking the OGs of C in G_N. If $G_N[C]$ is a complete graph (i.e. a graph where all vertices/OGs are connected to all other vertices), this implies that all OGs of C form a true microsyntenic block in N. If not, we can isolate the true microsyntenic blocks contained within $G_N[C]$ by retrieving all maximal cliques (i.e. subsets of

vertices that are all connected to each other and that cannot be expanded by including one more adjacent vertex).

Next, SYNPHONI sorts the maximal cliques of a given $G_N[C]$ by size and iterates through them starting with the largest. In order for a maximal clique to be retained as a true microsyntenic block, it must meet one the following conditions:

- The maximal clique comprises at least three vertices/OGs
- The maximal clique does not share OGs with an already saved maximal clique
- The maximal clique shares no more than one OG with no more than one already saved maximal clique

Cliques comprising only one or two vertices are also saved, if they include at least one self edge in G_{TN}, since they might correspond to microsyntenic blocks of N that comprise only paralogous genes. Each maximal clique retained after step 3 corresponds to an OG set inferred to form a true microsyntenic block of N (denoted S_{OG}).

STEP 4. Retrieve microsyntic blocks of extant species

Step 3 of the SYNPHONI pipeline generates sets of OGs, which correspond to "true" microsyntenic blocks inferred to have been present in a node of interest (N). The final step is to retrieve the descendants of these ancestrally microsyntenic sets of OGs (S_{OG}) in extant species, regardless of the amount of intervening genes (scale-free detection). The output produced by SYNPHONI can thus be used to describe the character states of syntenic blocks across vast evolutionary distances (e.g. species-/clade-specific loss of ancestral microsynteny with maintained macrosynteny, see examples in Figure 1 of the main article).

This step requires all S_{OG} (OG sets corresponding to true microsyntenic blocks of N) from step 3 and all GT_N (trimmed ancestral OG networks) used to identify them from step 2.5. First, all DNA strands (scaffolds/chromosomes) bearing members of the OGs contained in a given S_{OG} are isolated from all sample species. However, only DNA strands that satisfy both of the following conditions are retained:

- DNA strands bear at least three members from any OG contained in the S_{OG}
- DNA strands bear members from at least 30% of the OGs contained in the S_{OG}

This ensures that only DNA strands with sufficient coverage of the S_{OG} are kept. Note, however, that DNA strands bearing a sufficient number of paralogs to OG members of an S_{OG} are also retained.

For every S_{OG} of a given N, a graph $G_S[S_{OG}]$ is built in which each vertex corresponds to one filtered DNA strand of a single species (Supplementary Figure 5A). This graph may contain multiple vertices per species, if the true microsyntenic block of N was duplicated or has been split (e.g. due to inter-/intra-chromosomal rearrangements or fragmented genome assembly) in these species (e.g. Supplementary

Figure 5A, species S2). An edge will be added between two vertices, if all the following conditions are met:

- Vertices correspond to DNA strands from two different species (no same-species comparisons)
- Detection of at least three “OG co-occurrences” (OG co-occurrence = OG of the S_{OG} with members in both vertices/on both DNA strands)
- OG co-occurrence detected for at least 50% of the OGs of the S_{OG} that have members on the smaller DNA strand (i.e. at least half of the OGs of the S_{OG} that have members on the smaller DNA strand also have members on the other DNA strand)

Co-occurring paralogs (i.e. multiple members of the same OG of the S_{OG} that occur on both DNA strands) are only counted as separate OG co-occurrences if they were ancestrally microsyntenic in N. To check this, a graph $GT_N[S_{OG}]$ is built, which corresponds to a sub-graph of the trimmed ancestral OG network of N (GT_N) (Supplementary Figure 5B,C). If an OG of the $GT_N[S_{OG}]$ possesses a self-edge, all corresponding paralogs in $G_S[S_{OG}]$ are considered as ancestrally microsyntenic in N and are counted as separate OG co-occurrences (Supplementary Figure 5B). However, if an OG of the $GT_N[S_{OG}]$ does not possess a self-edge (due to absence of ancestrally microsyntenic paralogs), all corresponding paralog co-occurrences in $G_S[S_{OG}]$ are only counted as a single OG co-occurrence (Supplementary Figure 5C).

Every edge in the $G_S[S_{OG}]$ graph signifies that microsynteny of the S_{OG} is (at least partially) conserved between the DNA strands of two species. In order to obtain multi-species blocks, we next isolate sets of vertices that are interconnected. The communities in $G_S[S_{OG}]$ are identified, using the clique percolation method (Palla *et al.*, 2005) with a clique size of three. Thus, vertices with less than two links are discarded. Since edges in $G_S[S_{OG}]$ only link DNA strands from different species, the resulting multi-species blocks will contain at least three species.

Finally, the **BlocksByNode.py** script (Supplementary Table 3) was used to confirm that the multispecies blocks detected by SYNPHONI do not include artifacts. The script reexamines the taxonomic composition of each multispecies block, in order to determine whether it was indeed present in N (for details see Supplementary Material section 1.3). The species_threshold values used for this can be found in Supplementary Table 2.

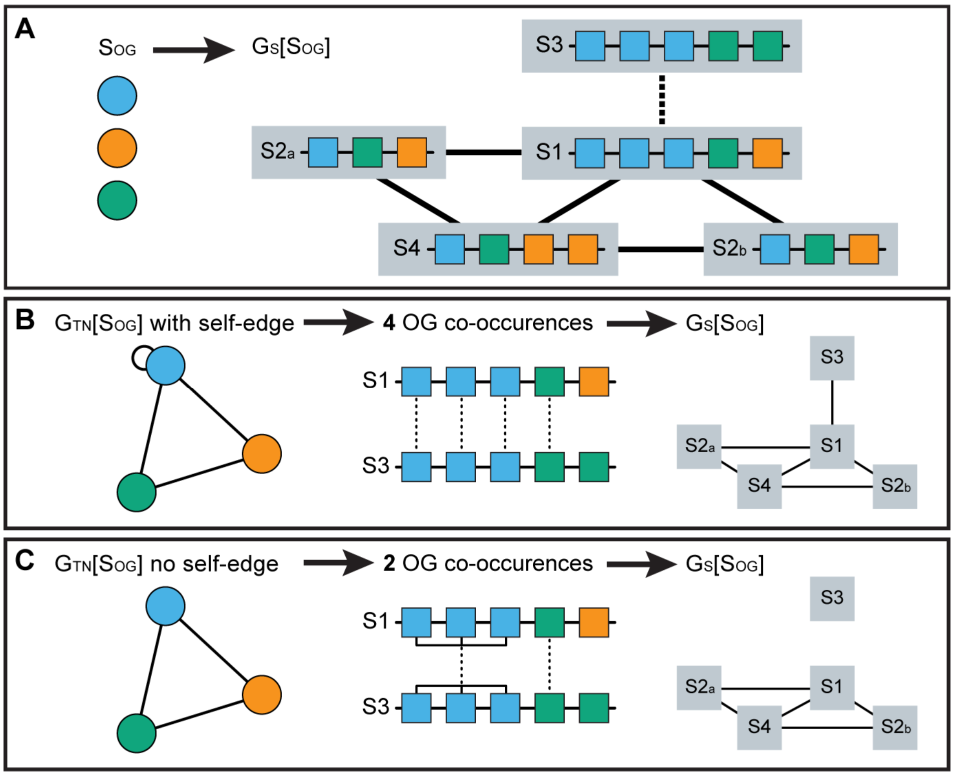

Supplementary Figure 5. Graphical summary of how OG occurrences are counted to infer conserved microsynteny between species pairs. (**A**) $G_S[S_{OG}]$ graph for an S_{OG} (true microsyntenic block of N) containing three different OGs (colored circles). Each vertex corresponds to a single DNA strand (scaffold/chromosome) from a single species (S1, S2, S3, S4). As per the conditions listed in step 4, only DNA strands bearing multiple members of the OGs contained in the S_{OG} (colored squares) are included. More than one vertex per species (e.g. S2a and S2b for species S2) signals that the true microsyntenic block of N has been split or duplicated in these species. An edge is only drawn between two vertices, if they are from different species (i.e. no same-species comparisons, e.g. S2a and S2b) and if a sufficient number of OG co-occurrences (OGs of the S_{OG} with members in both vertices) is detected. Thus, edges signify (at least partially) conserved microsynteny of the S_{OG} between the DNA strands of two species. The dotted edge signifies that between S1 and S3 conserved microsynteny of the S_{OG} may or may not be inferred, depending on whether the co-occurring paralogs (three blue squares = multiple members of the same OG of the S_{OG} that occur on both DNA strands) are ancestrally microsyntenic in N. (**B**) Scenario where the blue OG of the $GT_N[S_{OG}]$ possesses a self edge, which signifies that it contains ancestrally microsyntenic paralogs. Each member of this OG that occurs in both vertices (co-occurring paralogs, blue squares) is hence counted as a separate OG co-occurrence (dotted lines). As a result, four OG-co-occurrences are counted, and conserved microsynteny of the S_{OG} is inferred between S1 and S3. (**C**) Scenario where blue OG of the $GT_N[S_{OG}]$ graph does not possess a self edge, which signifies the absence of ancestrally microsyntenic paralogs. All members of the blue OG that occur in both vertices (co-occurring paralogs, blue squares) are hence counted as only a single OG co-occurrence (dotted lines). As a result, only two OG-co-occurrences are counted, and conserved microsynteny of the S_{OG} is not inferred between S1 and S3.

STEP 4.5 Count intervening genes

The script **analysis_intervening_genes.py** can be used to obtain the numbers of intervening genes between all consecutive members of each species block outputted by SYNPHONI. The nmax parameter specified in step3 restricts the maximal intergenic distance between OGs inferred to be ancestrally syntenic in a given node. However, the corresponding extant species blocks are retrieved regardless of whether gene proximity has been maintained or not. Thus, intergenic distances within a species block detected by SYNPHONI can vary from 0 to the maximal number of genes on the same scaffold/chromosome minus one. Nonetheless, the majority of consecutive block members show a distance of less than 10 intervening genes (Supplementary Figure 6). Intergenic distances between paralogs are generally lower than between non-paralogs, suggesting stronger evolutionary constrains on paralogous gene proximity. Blocks detected for more recent nodes (e.g. Cephalopoda, Protostomia) also show lower intergenic distances than those detected for more ancient nodes (e.g. Parahoxozoa, Metazoa). This is likely because genomic reshuffling increases with divergence time. Consistently, nmax (inferred maximal ancestral intergenic distance) marks the descending inflexion point of the curves describing the intergenic distance distributions of each node (Supplementary Figure 6).

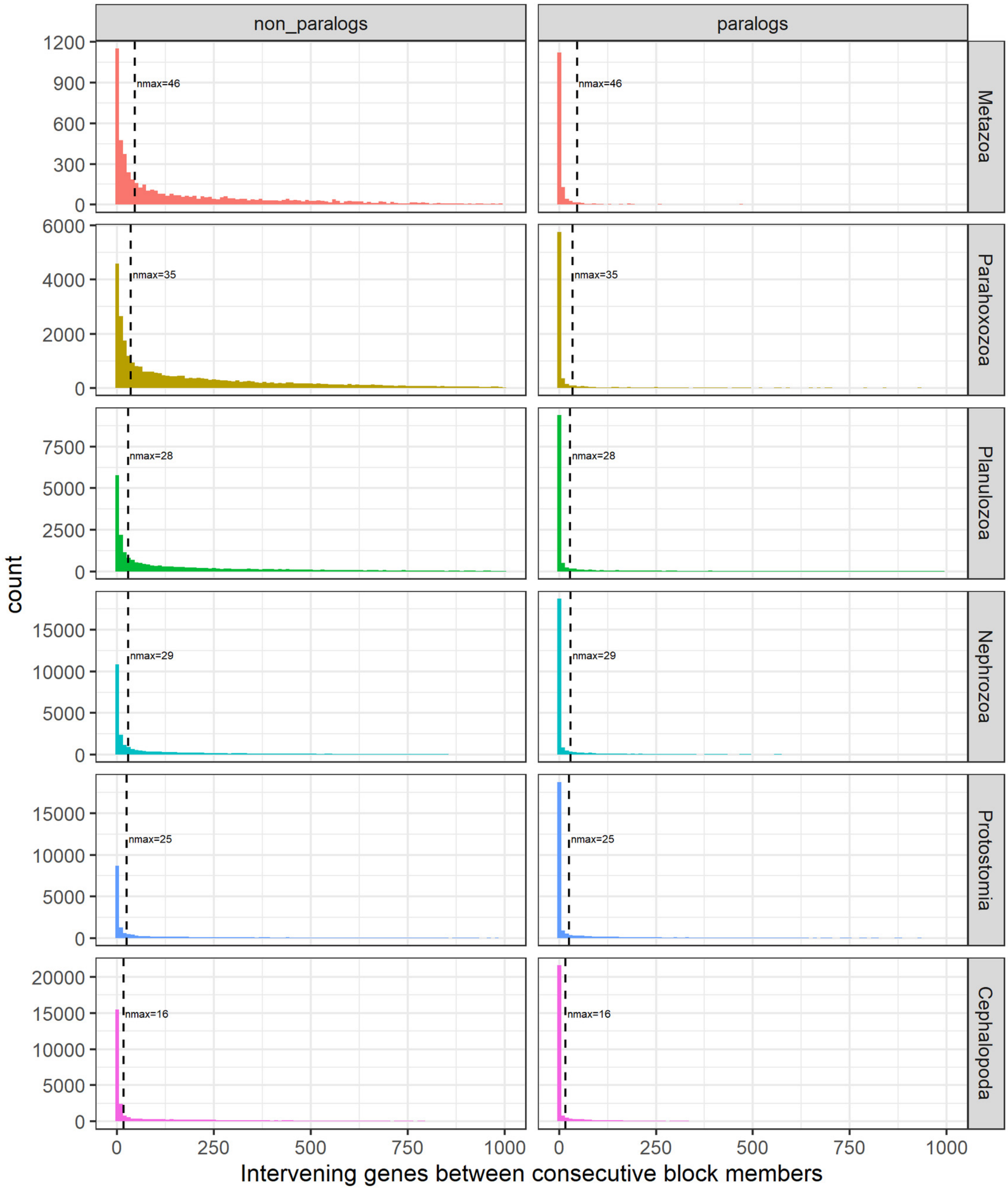

Supplementary Figure 6. Distribution of intergenic distances between consecutive members of the species blocks detected by SYNPHONI. The dotted line indicates the maximal intergenic distance (nmax) threshold used for the ancestral microsyntenic block inference in step3. Distances greater than 1000 intervening genes were omitted for readability purposes (corresponds to 2.2 - 4.4% of all values per

node). The highest intergenic distance measured was 4615 intervening genes in *C. elegans* (Parahoxozoa node).

3. Benchmarks

In order to evaluate the microsyntenic blocks detected by SYNPHONI, we compared them to those detected by the MicroSynteny tool (Simakov *et al.*, 2013) and EvolClust (Marcet-Houben and Gabaldón, 2019). All three methods are able to infer non-collinear multispecies blocks that are conserved across numerous genomes. Each analysis was performed using our extensive taxon sampling of 80 metazoan genomes (Supplementary Table 1).

First, we tested whether the detected multispecies blocks are evolutionarily meaningful, by assessing whether they are chiefly composed of "core OGs" (see Section 1.4 and **describe_OGs.py** script, Supplementary Table 3). All the blocks detected by SYNPHONI and EvolClust as well as the vast majority of blocks detected by the MicroSynteny tool comprise at least one core OG (Supplementary Figure 7A). However, calculating the noise level within the detected blocks (i.e. number of core OGs per block in a given block/total number of OGs per block) shows that SYNPHONI produces the least noise out of the three methods (Supplementary Figure 7B).

Both EvolClust and the MicroSynteny tool output only a single set of blocks for all species analyzed, whereas SYNPHONI identifies the multispecies blocks of all six nodes of interest (N, Supplementary Figure 1) separately. We thus assigned the blocks produced by EvolClust and the MicroSynteny tool to their corresponding Ns, based on their taxonomic composition (see Section 1.3 and **BlocksByNode.py** script, Supplementary Table 3). To evaluate block detection sensitivity, we then compared the number of species in which homologs to a given multispecies block were found (i.e. number of species per multispecies block, Supplementary Figure 7C). For all nodes considered, SYNPHONI detects the most homologs by a good margin. This is likely because SYNPHONI identifies blocks in a scale-free manner (i.e irrespective of taxon-specific changes to intergenic distances between syntenic OGs).

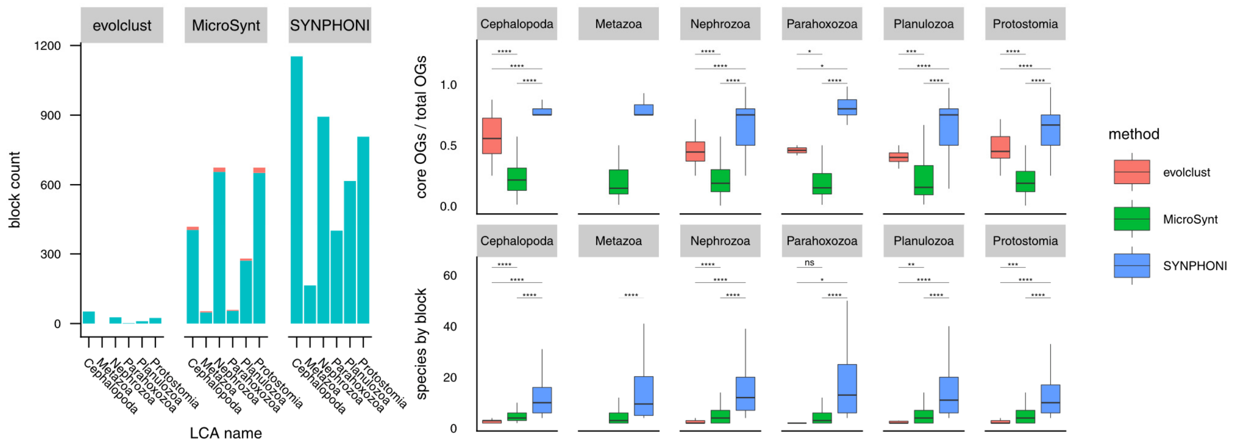

Supplementary Figure 7. Benchmark results of SYNPHONI versus the MicroSynteny tool and EvolClust. (**A**) Number of multispecies blocks assigned to each node of interest (N). The colors in the stacked histogram correspond to blocks with at least one core OG (blue), or with no core OG (red). (**B**) The noise level of blocks recovered by EvolClust (evolclust, red), the MicroSynteny tool (MicroSynt, green) or SYNPHONI (blue) is represented by the proportion of core OGs (see supplementary figure 5) to the total number of OGs present within each multispecies block. (**C**) The sensitivity of each method is represented by the number of species per multispecies block. The box plots indicate the interquartile range and median of each distribution. The whiskers indicate the furthest sample within 1.5 times the interquartile range. The p-values were determined using two-sided Wilcoxon rank-sum tests to compare the bracketed distributions (*:p < 0.05; **:p < 0.01; ***: p < 0.001; ****: p < 0.0001).

In addition, we determined whether all three methods return similar blocks. To this end, we built a graph G_B where each vertex corresponds to a multispecies block from a given method. Edges were only drawn between multispecies blocks from different methods, if they comprised similar sets of core OGs (Jaccard index > 0.1). Each connected component of the G_B graph was then isolated and counted as a single "block occurrence". Thus, every block occurrence corresponded to a multispecies block that was detected by at least one and up to three methods. Out of the total number of block occurrences detected in each N, SYNPHONI recovered 99.3% (140/141) in Metazoa, 99.1% (349/352) in Parahoxozoa, 96.7% (429/444) in Planulozoa, 90.9% (499/549) in Nephrozoa, 83% (366/441) in Protostomia and 98.5% (939/952) in Cephalopoda (Supplementary Figure 8). Moreover, depending on the node, 47.8% to 97.2% of the blocks were SYNPHONI-specific.

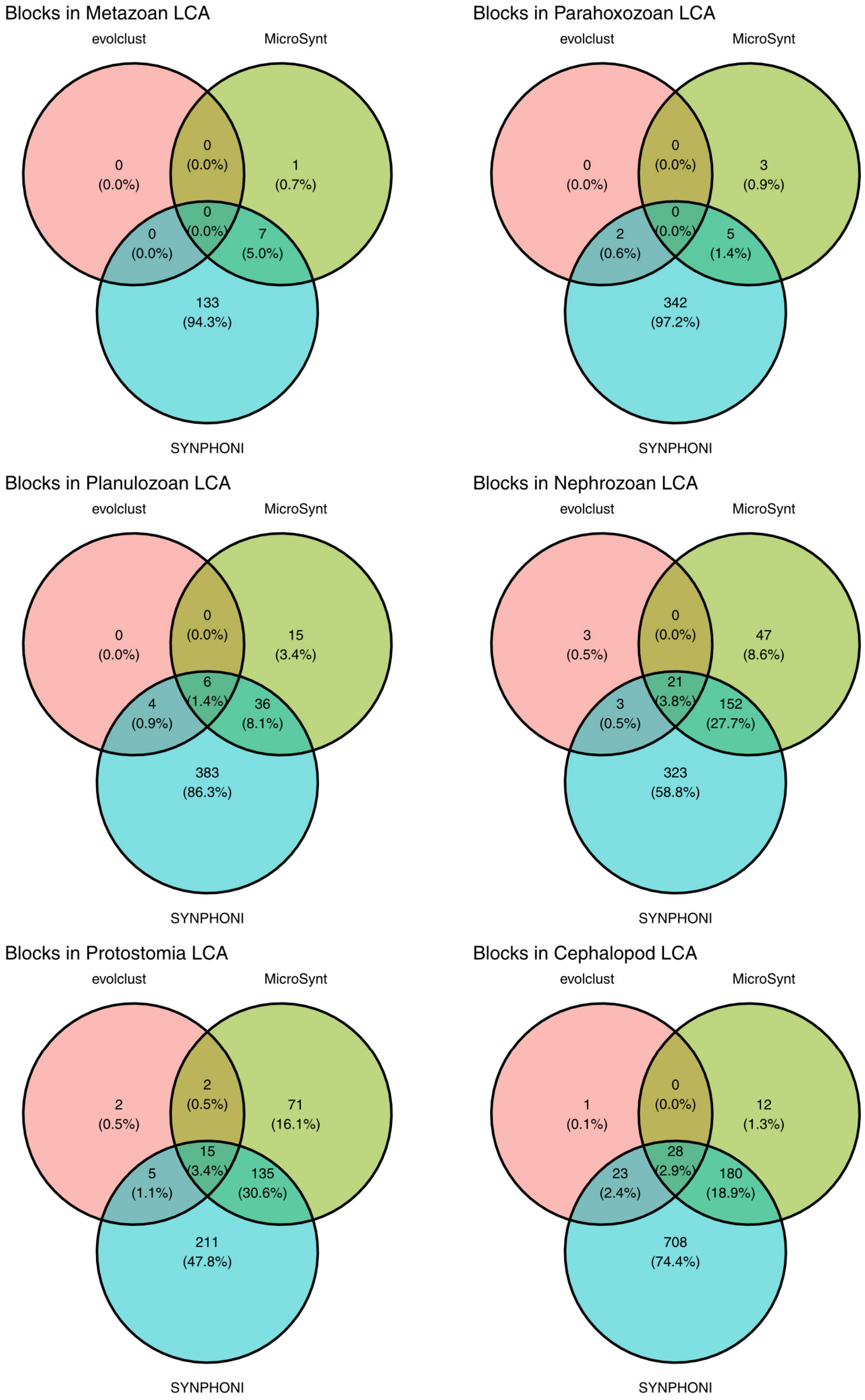

Supplementary Figure 8. Venn diagrams comparing the number of block occurrences detected by SYNPHONI (blue), EvolClust (red) and/or the MicroSynteny tool (green) for each node of interest.

Of note, EvolClust detected less multispecies blocks (Supplementary Figure 7A) and single block occurrences (Supplementary Figure 8) than the other methods, regardless of the node considered (Supplementary Figure 7A, Supplementary Figure 8). This is likely because we supplied EvolClust with an orthology that was not at gene family but at orthogroup level, leading to a more stringent definition of significant gene clusters (Marcet-Houben and Gabaldón, 2019). Furthermore, SYNPHONI and the MicroSynteny tool can detect blocks composed of paralogs, whereas EvolClust only infers blocks composed of genes that originate from different gene families. In sum, these analyses show that SYNPHONI is not only less prone to noise detection but also more sensitive than other comparable methods across all taxonomic nodes analyzed.

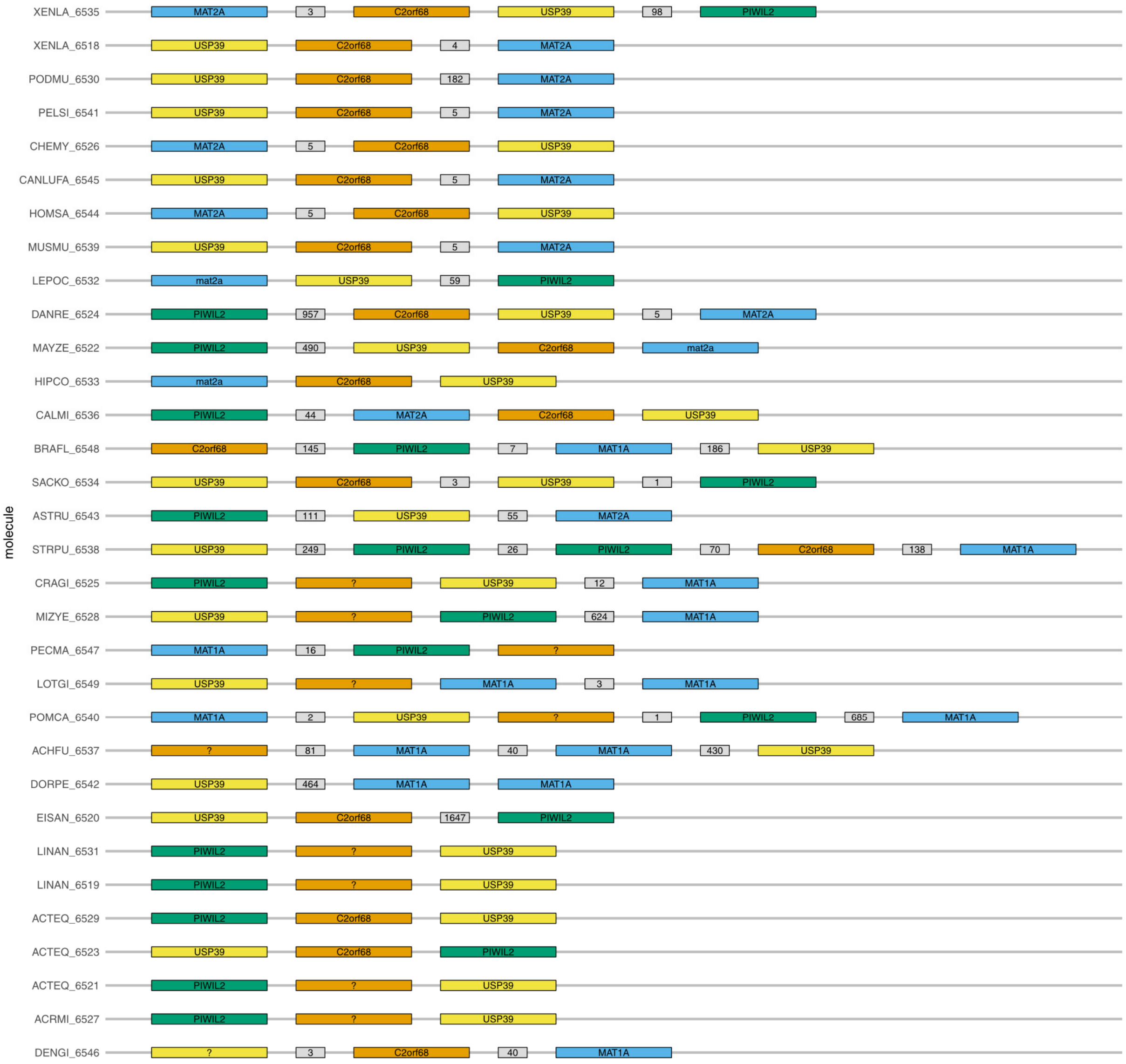

Supplementary Figure 9. Graphical summary of the nephrozoan PWIL2 block, as detected by SYNPHONI. Colored rectangles represent genes of the PIWI2L ancestral microsyntenic block that were recovered as syntenic in the sample species. The species block names (left) are composed of the five-letter species prefix (see Supplementary Table 1 for the full binomial names) and the four-digit block ID. The color of a rectangle indicates its respective OG identity. Within each rectangle the putative gene name, as predicted by eggNOG-mapper, is provided. Interrogation marks signify that no gene name could be predicted. According to our annotation, the depicted OGs correspond to the following orthologs: PIWI2L (green), C2orf68 (orange), USP39 (yellow) and MAT1A/MAT2A (blue). Gray rectangles indicate the presence and number of intervening genes between gene members of a species block.

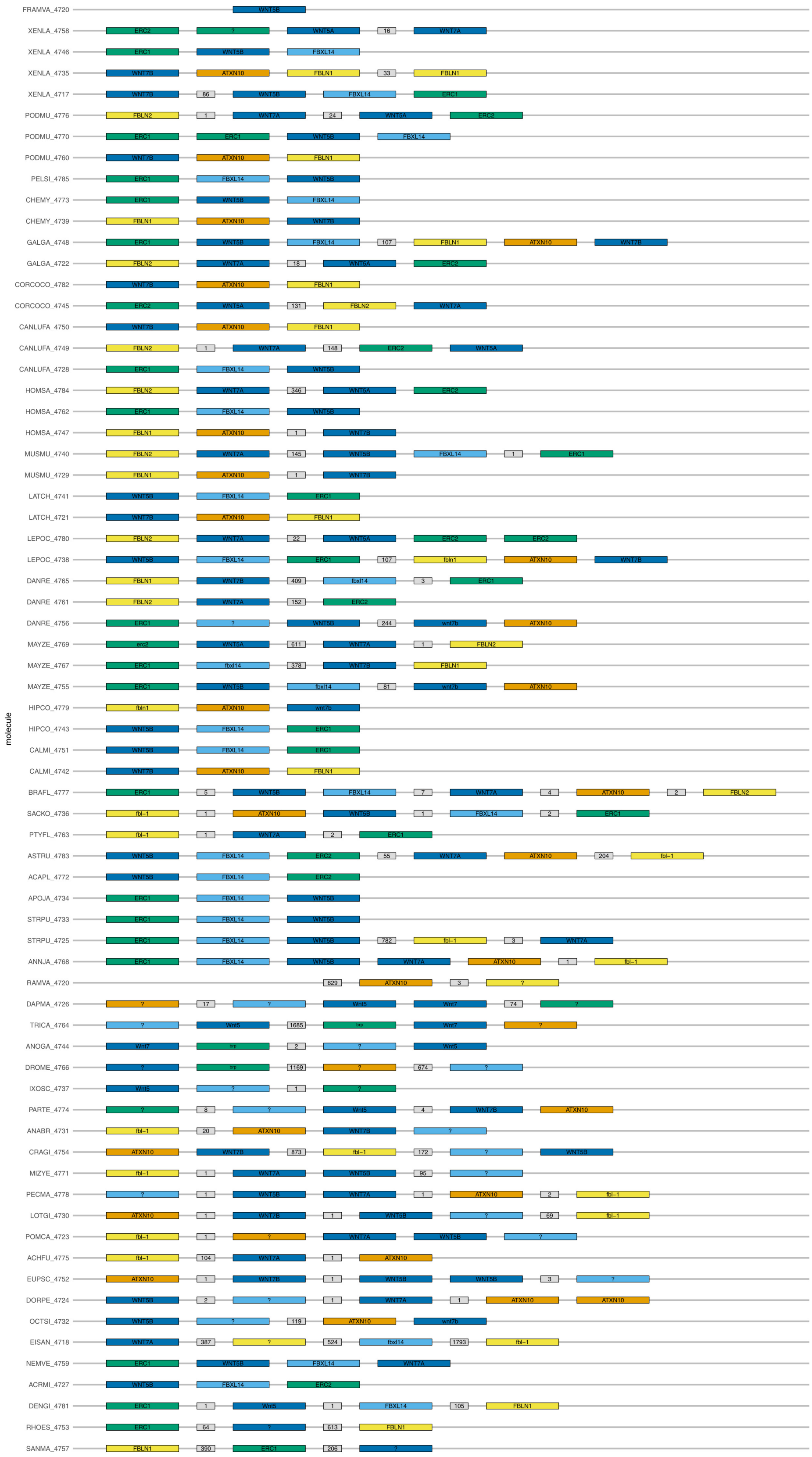
FRAMVA_4720
XENLA_4758
XENLA_4746
XENLA_4735
XENLA_4717
PODMU_4776
PODMU_4770
PODMU_4760
PELSI_4785
CHEMY_4773
CHEMY_4739
GALGA_4748
GALGA_4722
CORCOCO_4782
CORCOCO_4745
CANLUFA_4750
CANLUFA_4749
CANLUFA_4728
HOMSA_4784
HOMSA_4762
HOMSA_4747
MUSMU_4740
MUSMU_4729
LATCH_4741
LATCH_4721
LEPOC_4780
LEPOC_4738
DANRE_4765
DANRE_4761
DANRE_4756
MAYZE_4769
MAYZE_4767
MAYZE_4755
HIPCO_4779
HIPCO_4743
CALMI_4751
CALMI_4742
BRAFL_4777
SACKO_4736
PTYFL_4763
ASTRU_4783
ACAPL_4772
APOJA_4734
STRPU_4733
STRPU_4725
ANNJA_4768
RAMVA_4720
DAPMA_4726
TRICA_4764
ANOGA_4744
DROME_4766
IXOSC_4737
PARTE_4774
ANABR_4731
CRAGI_4754
MIZYE_4771
PECMA_4778
LOTGI_4730
POMCA_4723
ACHFU_4775
EUPSC_4752
DORPE_4724
OCTSI_4732
EISAN_4718
NEMVE_4759
ACRMI_4727
DENGI_4781
RHOES_4753
SANMA_4757
molecule

Supplementary Figure 10. Graphical summary of the nephrozoan WNT5/7 block, as detected by SYNPHONI. Colored rectangles represent genes of the WNT5/7 ancestral microsyntenic block that were recovered as syntenic in the sample species. The species block names (left) are composed of the five-letter species prefix (see Supplementary Table 1 for the full binomial names) and the four-digit block ID. The color of a rectangle indicates its OG identity. Putative gene names, as assigned by eggNOG-mapper, are provided within each rectangle. Interrogation marks signify that no gene name could be predicted. According to our annotation, the depicted OGs correspond to the following orthologs: ERC1/ERC2 (green), ATXN10 (orange), FBXL14 (light blue), FBLN1/FBLN2 (yellow), and WNT5/WNT7 (dark blue). Gray rectangles indicate the presence and number of intervening genes between the gene members of a species block.Of note, microsynteny of spiralian ERC1/2 orthologs has previously been described, based on manual annotation of the WNT5/7 block (Robert *et al.*, 2022).It was, however, not detected by SYNPHONI, because the spiralian ERC1/2 orthologs were assigned to a different OG in our OrthoFinder run.

4. Evaluation of ancestral network inference using simulated data (ZOMBI-SYNPHONI)

To test how accurately SYNPHONI infers ancestral synteny, it was run on simulated data. For this we used the ZOMBI tool (Davín et al. 2020) with default settings, except:

- initial ancestral genome size was set to 500 genes (to simulate the evolution of a single chromosome)
- number of extant lineages was set to 80 (same as the taxonomic sample used in this study)
- minimum size of extant genomes was set to 250 genes (to prevent excessive gene loss)
- horizontal gene transfer rate was set to zero (since SYNPHONI is intended for animal genome analysis)

Comparing more ancient nodes (e.g. n4, n7, n8) with more recent nodes (e.g. n45, n62) (Supplementary Figure 11) shows that genomic reshuffling increases with divergence time (Supplementary Figure 12, Oxford dot plots). Ancestral genome reconstruction methods requiring collinearity conservation are thus not very suitable for distantly related taxa. In contrast, SYNPHONI-inferred ancestral intergenic distances show a strong positive correlation with the actual ZOMBI-simulated ancestral distances across all nodes (Supplementary Figure 12, 2D Kernel Density plots and linear regression statistics), with only minimal noise on the most ancient node (n4). This confirms that the ancestral OG networks built by SYNPHONI are highly accurate across all evolutionary time-scales.

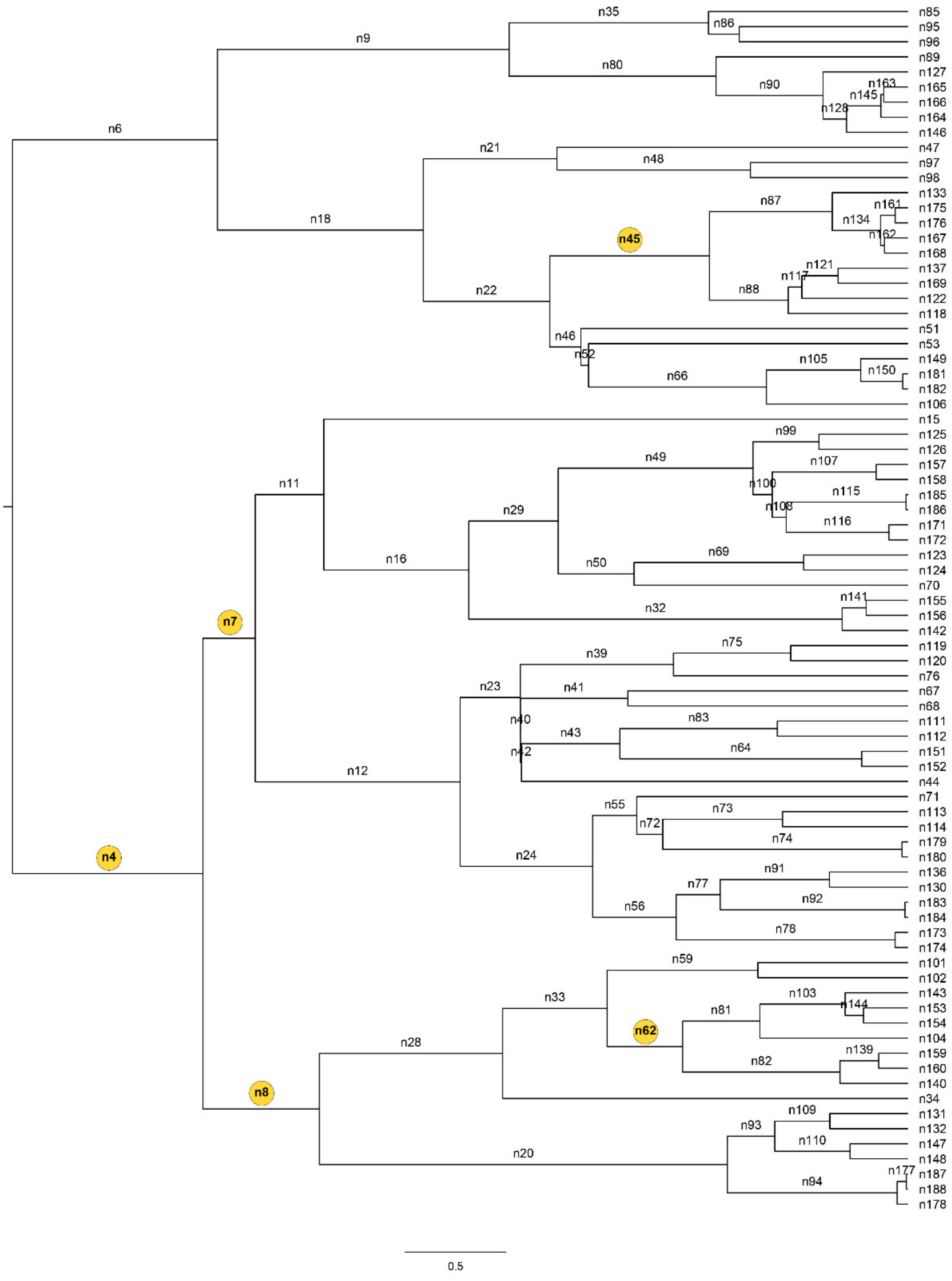

Supplementary Figure 11. Cladogram of the phylogenetic relationships between ZOMBI-simulated genomes. Nodes used for further analysis are highlighted by a yellow circle.

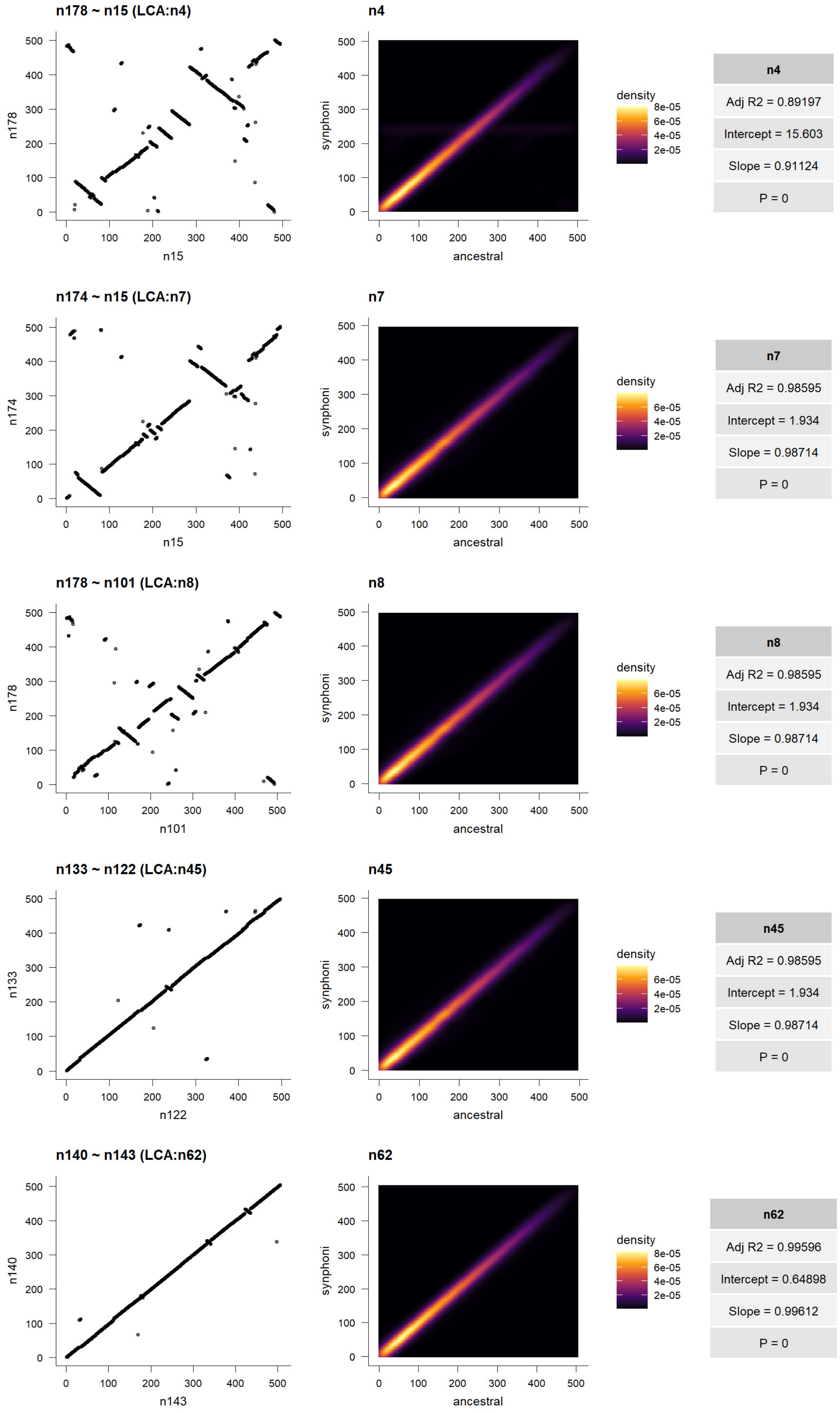

n178 ~ n15 (LCA:n4)
n178
n15
n4
synphoni
ancestral
density
8e-05
6e-05
4e-05
2e-05
n4
Adj R2 = 0.89197
Intercept = 15.603
Slope = 0.91124
P = 0
n174 ~ n15 (LCA:n7)
n174
n15
n7
synphoni
ancestral
density
6e-05
4e-05
2e-05
n7
Adj R2 = 0.98595
Intercept = 1.934
Slope = 0.98714
P = 0
n178 ~ n101 (LCA:n8)
n178
n101
n8
synphoni
ancestral
density
6e-05
4e-05
2e-05
n8
Adj R2 = 0.98595
Intercept = 1.934
Slope = 0.98714
P = 0
n133 ~ n122 (LCA:n45)
n133
n122
n45
synphoni
ancestral
density
6e-05
4e-05
2e-05
n45
Adj R2 = 0.98595
Intercept = 1.934
Slope = 0.98714
P = 0
n140 ~ n143 (LCA:n62)
n140
n143
n62
synphoni
ancestral
density
8e-05
6e-05
4e-05
2e-05
n62
Adj R2 = 0.99596
Intercept = 0.64898
Slope = 0.99612
P = 0

Supplementary Figure 12. Synteny conservation across ZOMBI-simulated genomes. Five nodes at different phylogenetic depths (n4, n7, n8, n45, n62) were analyzed (see also Supplementary Figure 11). Oxford dot plots (left) compare orthologous gene locations between two descendants (e.g. n178 ~ n15) of a given node or "last common ancestor" (e.g. LCA:n4). Low correspondence of ortholog locations between two extant genomes indicates an extensive loss of collinearity (e.g. n4, n7, n8). 2D Kernel Density plots (middle) compare the ancestral intergenic distances between syntenic OG pairs that were inferred by SYNPHONI (synphoni, y-axis) to those that were actually simulated by ZOMBI (ancestral, x-axis). Color-scales indicate the density of data points. The linear regression statistics (left) confirm that the ZOMBI-simulated and the SYNPHONI-inferred intergenic distances show a strong and highly significant positive correlation, with R-squared (R2) > 0.89 and a p-value (P) < 1e-15 (could only be outputted as "0").

References

Albalat,R. and Cañestro,C. (2016) Evolution by gene loss. Nature Reviews Genetics, 17, 379–391.

Buchfink,B. *et al.* (2015) Fast and sensitive protein alignment using DIAMOND. *Nat. Methods*, **12**, 59–60.

Cannon,J.T. *et al.* (2016) Xenacoelomorpha is the sister group to Nephrozoa. *Nature*, **530**, 89–93.

Chiari,Y. *et al.* (2012) Phylogenomic analyses support the position of turtles as the sister group of birds and crocodiles (Archosauria). *BMC Biol*, **10**, 65.

Christopoulos,D.T. (2016) On the Efficient Identification of an Inflection Point. **6**, 8.

Davín, A.A. et al. (2020) Zombi: a phylogenetic simulator of trees, genomes and sequences that accounts for dead lineages. *Bioinformatics*, **36**(4), 1286-1288.

Dewey,C.N. (2011) Positional orthology: putting genomic evolutionary relationships into context. *Briefings in Bioinformatics*, **12**, 401–412.

Dunn,C.W. *et al.* (2014) Animal Phylogeny and Its Evolutionary Implications. *Annu Rev Ecol Evol Syst*, **45**, 371–395.

Emms,D.M. and Kelly,S. (2019) OrthoFinder: phylogenetic orthology inference for comparative genomics. *Genome Biol.*, **20**, 238.

Fernández,R. and Gabaldón,T. (2020) Gene gain and loss across the metazoan tree of life. *Nat Ecol Evol*, **4**, 524–533.

Giribet,G. and Edgecombe,G.D. (2017) Current Understanding of Ecdysozoa and its Internal Phylogenetic Relationships. *Integrative and Comparative Biology*, **57**, 455–466.

Huerta-Cepas,J. *et al.* (2017) Fast Genome-Wide Functional Annotation through Orthology Assignment by eggNOG-Mapper. *Mol. Biol. Evol.*, **34**, 2115–2122.

Katoh,K. and Standley,D.M. (2013) MAFFT Multiple Sequence Alignment Software Version 7: Improvements in Performance and Usability. *Mol. Biol. Evol.*, **30**, 772–780.

Kayal,E. *et al.* (2018) Phylogenomics provides a robust topology of the major cnidarian lineages and insights on the origins of key organismal traits. *BMC Evol Biol*, **18**, 68.

Leclère,L. *et al.* (2019) The genome of the jellyfish *Clytia hemisphaerica* and the evolution of the cnidarian life-cycle. *Nat. Ecol. Evol.*, **3**, 801–810.

Marcet-Houben,M. and Gabaldón,T. (2019) EvolClust: automated inference of evolutionary conserved gene clusters in eukaryotes. *Bioinformatics*, btz706.

Marlétaz,F. *et al.* (2019) A New Spiralian Phylogeny Places the Enigmatic Arrow Worms among

Gnathiferans. *Current Biology*, **29**, 312-318.e3.
Mendivil Ramos,O. and Ferrier,D.E.K. (2012) Mechanisms of Gene Duplication and Translocation and Progress towards Understanding Their Relative Contributions to Animal Genome Evolution. *International Journal of Evolutionary Biology*, **2012**, 1–10.
Murgarella,M. *et al.* (2016) A First Insight into the Genome of the Filter-Feeder Mussel Mytilus galloprovincialis. *PLOS ONE*, 22.
Palla,G. *et al.* (2005) Uncovering the overlapping community structure of complex networks in nature and society. **435**, 5.
Paps,J. and Holland,P.W.H. (2018) Reconstruction of the ancestral metazoan genome reveals an increase in genomic novelty. *Nat Commun*, **9**, 1730.
Pevzner,P. and Tesler,G. (2003) Genome Rearrangements in Mammalian Evolution: Lessons From Human and Mouse Genomes. *Genome Research*, **13**, 37–45.
Price,M.N. *et al.* (2010) FastTree 2 – Approximately Maximum-Likelihood Trees for Large Alignments. *PLoS ONE*, **5**, e9490.
Reich,A. *et al.* (2015) Phylogenomic Analyses of Echinodermata Support the Sister Groups of Asterozoa and Echinozoa. *PLoS ONE*, **10**, e0119627.
Robert,N.S.M. *et al.* (2022) Emergence of distinct syntenic density regimes is associated with early metazoan genomic transitions. *BMC Genomics*, **23**, 143.
Sacerdot,C. *et al.* (2018) Chromosome evolution at the origin of the ancestral vertebrate genome. *Genome Biol.*, **19**, 166.
Simakov,O. *et al.* (2022) Deeply conserved synteny and the evolution of metazoan chromosomes. *Sci. Adv.*, **8**, eabi5884.
Simakov,O. *et al.* (2013) Insights into bilaterian evolution from three spiralian genomes. *Nature*, **493**, 526–531.
Srivastava,M. *et al.* (2010) The *Amphimedon queenslandica* genome and the evolution of animal complexity. *Nature*, **466**, 720–726.
Torruella,G. *et al.* (2012) Phylogenetic Relationships within the Opisthokonta Based on Phylogenomic Analyses of Conserved Single-Copy Protein Domains. *Molecular Biology and Evolution*, **29**, 531–544.
Van Dongen,S. (2008) Graph Clustering Via a Discrete Uncoupling Process. *SIAM J. Matrix Anal. & Appl.*, **30**, 121–141.
Winter,S. *et al.* (2016) Finding approximate gene clusters with Gecko 3. *Nucleic Acids Res*, gkw843.
Wong,W.Y. *et al.* (2019) Expansion of a single transposable element family is associated with genome-size increase and radiation in the genus Hydra. *Proceedings of the National Academy of Sciences of the United States of America*, 3.
